# Exploring the Chemistry and Applications of Thio-, Seleno-, and Tellurosugars

**DOI:** 10.3390/molecules30092053

**Published:** 2025-05-05

**Authors:** Roxana Martínez-Pascual, Mario Valera-Zaragoza, José G. Fernández-Bolaños, Óscar López

**Affiliations:** 1 Centro de Investigaciones Científicas, Instituto de Química Aplicada, Universidad del Papaloapan, Circuito Central 200, Col. Parque Industrial, Tuxtepec 68301, Oaxaca, Mexico; rpascual@unpa.edu.mx (R.M.-P.); mvalera@unpa.edu.mx (M.V.-Z.); 2 Departamento de Química Orgánica, Facultad de Química, Universidad de Sevilla, Apartado 1203, E-41071 Seville, Spain; bolanos@us.es

**Keywords:** glycomimetics, thiosugars, selenosugars, tellurosugars, thioglycosides, selenoglycosides

## Abstract

Given the crucial roles of carbohydrates in energy supply, biochemical processes, signaling events and the pathogenesis of several diseases, the development of carbohydrate analogues, called glycomimetics, is a key research area in Glycobiology, Pharmacology, and Medicinal Chemistry. Among the many structural transformations explored, the replacement of endo- and exocyclic oxygen atoms by carbon (carbasugars) or heteroatoms, such as nitrogen (aza- and iminosugars), phosphorous (phosphasugars), sulfur (thiosugars), selenium (selenosugars) or tellurium (tellurosugars) have garnered significant attention. These isosteric substitutions can modulate the carbohydrate bioavailability, stability, and bioactivity, while introducing new properties, such as redox activity, interactions with pathological lectins and enzymes, or cytotoxic effects. In this manuscript we have focused on three major families of glycomimetics: thio-, seleno-, and tellurosugars. We provide a comprehensive review of the most relevant synthetic pathways leading to substitutions primarily at the endocyclic and glycosidic positions. The scope includes metal-catalyzed reactions, organocatalysis, electro- and photochemical transformations, free-radical processes, and automated syntheses. Additionally, mechanistic insights, stereoselectivity, and biological properties are also discussed. The structural diversity and promising bioactivities of these glycomimetics underscore their significance in this research area.

## 1. Introduction

Carbohydrates are ubiquitous biomolecules with essential biological functions across all domains of life. Beyond serving as a primary energy source and storage form, they play crucial structural roles (e.g., cellulose, chitin), and act as key building blocks in anabolic pathways, including nucleic acids biosynthesis [1]. Complex carbohydrates (glycans) [1] are also integral components of glycoconjugates such as glycolipids and glycoproteins, which are embedded in cell membranes and participate in critical signaling events, both endogenous and pathogen-mediated.

Moreover, carbohydrates serve as templates for the development of novel therapeutics [1] targeting metabolic disorders [2], cancer (e.g., carbohydrate-based vaccines) [3], infectious diseases [4], and drug delivery systems, including nanocarriers [5].

**Figure 1:**
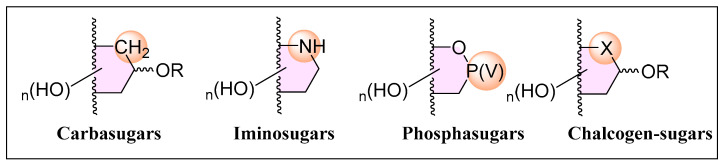
General structure of relevant glycomimetics.

Given their immense biological significance, carbohydrate analogues, referred to as glycomimetics, have emerged as a promising research area, offering deeper insights into carbohydrate interactions, modulating bioactivity, and even introducing novel properties. Among the vast array of synthetic modifications applicable to carbohydrate structures, bioisosteric replacements of oxygen and carbon atoms deserve attention. The substitution of the endocyclic oxygen, or the anomeric carbon with carbon (carbasugars) [6], nitrogen (iminosugars) [7], phosphorous (phosphasugars) [8], sulfur (thiosugars) [9], selenium (selenosugars) [10] and tellurium (tellurosugars) [11] (Figure 1) has led to the development of an arsenal of compounds that have expanded the frontiers of Glycobiology research, and constitute some of the most relevant glycomimetics.

In this review, we will discuss recent advances in the development of chalcogen-containing glycomimetics and their implication in Glycoscience.

## 2. S-Containing Carbohydrates

**Figure 2:**
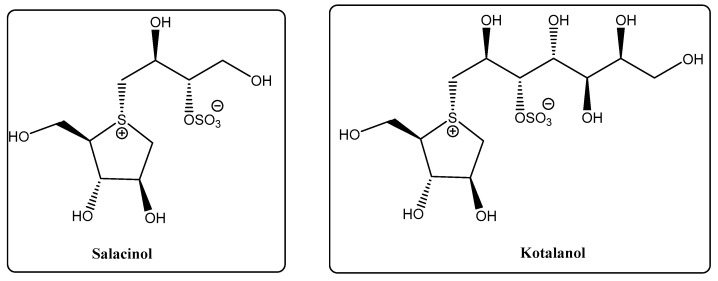
Structure of salacinol and kotalanol.

Thiosugars represent a vast family of glycoconjugates in which either a hydroxyl group or the endocyclic oxygen is replaced by sulfur. This isosteric substitution induces significant conformational and physico-chemical changes in the sugar mimic. Due to its bigger atomic radius, sulfur forms longer C–S bonds compared to oxygen, resulting in smaller dihedral angles [12]. Additionally, sulfur has smaller electronegativity and a more hydrophobic character, while not exhibiting hydrogen bonding acceptance capacity, which affects water solubility [13]; the stronger sulfur-π interactions between thiosugars and lectins compared to oxygen can be useful in the design of effective drugs [14]. Many thiosugars exhibit substantially improved hydrolytic or enzymatic stability compared to their oxygen-containing counterparts, which is undoubtedly advantageous for the design of new drugs [9]. There are some examples found in nature, like 5-thio-d-mannose, thioglycosides (e.g., glucosinolates) and 1,4-thioanhydrosugars (e.g., kotalanol, salacinol, Figure 2) [15].

### 2.1. 4′- and 5′-Thiosugars

Classical synthetic approaches for introducing a sulfur atom into a ring to give thiosugars have been recently reviewed [16]; one of the most successful approaches involves the introduction of a leaving group at the appropriate position of the sugar, and reaction with a sulfur-containing nucleophile. Herein, recent syntheses of thiosugars with practical interest will be reviewed.

**Scheme 1:**
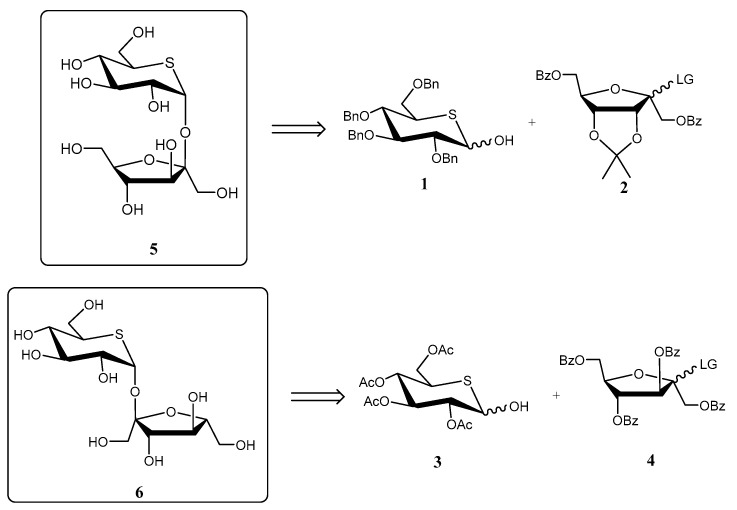
Retrosynthetic analysis for the preparation of (+)-5-thiososucrose (**5**) and (+)-5-thioisosucrose (**6**).

Ueda and co-workers reported [17] the preparation of the sucrose analogues (+)-5-thiosucrose (**5**) and (+)-5-thioisosucrose (**6**) as potential sweeteners and glycosidase inhibitors. The key step of the synthesis was a stereoselective glycosylation reaction between a d-psicose or a d-fructose donor, respectively, and a 5-thio-d-glucose derivative (**1** and **3**) as acceptors (Scheme 1). Due to the strong anomeric effect induced by **1** and **3**, they acted as α-directing glycosyl acceptors. Unfortunately, none of the compounds displayed appreciable activity as sweeteners or inhibitors [17].

**Scheme 2:**
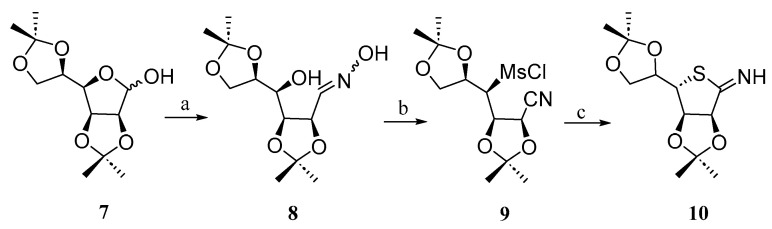
Synthesis of thiomonosaccharide **10**. Reagents and conditions: (a) NH_2_OH·HCl, NaHCO_3_; (b) MsCl, 0 °C→r.t.; (c) Na_2_S·9H_2_O, DMF.

Díaz-Fernández and Pino-González reported [18] a simple approach for the synthesis of the thiomonosaccharide **10** from d-mannose. The synthesis involved the use of di-*O*-isopropylidene d-mannose **7**, which was transformed into the diastereomeric *E*/*Z* mixture of oxime **8** using hydroxylamine hydrochloride under basic conditions. The hydroxyl groups of **8** were subsequently mesylated to produce compound **9**. Finally, the mesylate group underwent an S_N_2 substitution reaction with Na_2_S, followed by intramolecular cyclization on the nitrile group, yielding the thiosugar **10** (Scheme 2).

**Scheme 3:**
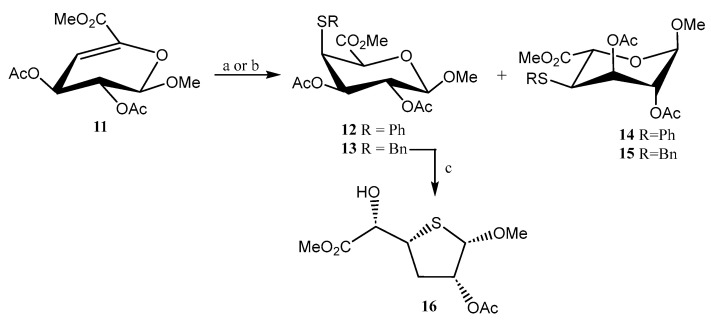
Synthesis of *S*-linked uronate derivatives and formation of the thiofuronoside **16**. Reagents and conditions: (a) PhSH, BnSH; (b) BnSH, thermal/photo-inititator; (c) AlCl_3_, toluene, 48 h.

Liu and coworkers explored [19] the preparation of *S*-linked uronates (**12**–**15**) using a thiolation reaction (PhSH, BnSH) under alkaline and radical-mediated conditions on Δ^4,5^-unsaturated methyl uronate **11** (Scheme 3). The reaction demonstrated to be inefficient under radical conditions, which was attributed to the electron-withdrawing effect of the ester moiety located on C-5, although it proceeded with good regio- and stereoselectivity at C-4 and C-5 positions. Under basic conditions, good stereoselectivity was obtained at C-4, but, conversely, poor stereoselectivity at C-5, tentatively due to a retro thiol-Michael reaction. Attempts to obtain a *S*-linked pseudodisaccharide failed as, unexpectedly, when **13** was treated with a Lewis acid (AlCl_3_) to remove the benzyl group, the thiofuranoside **16** was obtained serendipitously (Scheme 3).

A family of thiosugars that has gained considerable attention is the sulfonium-containing carbohydrates, as they are analogues of the naturally occurring kotalanol and salacinol (Figure 2), both isolated from *Salacia reticulata* extracts, and known to be potent inhibitors of intestinal glycosidases [20]. These sulfonium glycomimetics are therefore promising candidates for the treatment of diabetes [21].

**Scheme 4:**
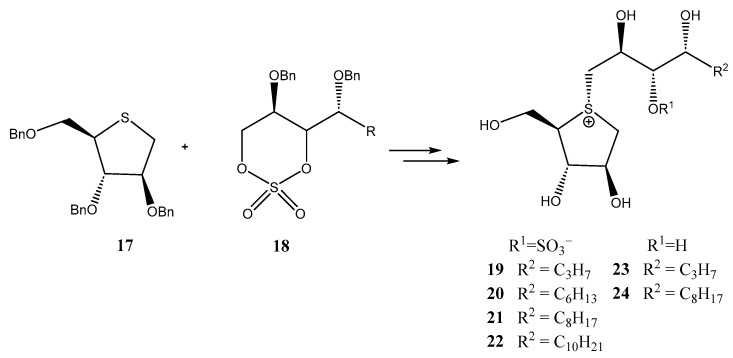
Preparation of salacinol derivatives with an elongated side chain.

In this context, Takashima et al. hypothesized [22] that the incorporation of a hydrophobic moiety into the side chain of salacinol could enhance its inhibitory activity against glycosidases through favorable van der Waals interactions. To test this hypothesis, derivatives **19**–**24** were prepared (Scheme 4), the alkylation of *O*-protected thiosugar **17** using cyclic sulfates **18** being the key step. The final compounds were evaluated as inhibitors of rat maltase, sucrose and isomaltase, as well as human maltase. While no clear effects of the hydrocarbon residue were observed on maltase or isomaltase inhibition, sucrose inhibition displayed a length-dependent inhibitory activity, with longer appendages yielding the most potent compounds (IC_50_ = 0.15–0.73 µM for **19**–**22**, 1.4 and 0.38 µM for **23** and **24**). This resulted in an up to ten-fold increase in activity compared to parent salacinol [22]. Additionally, de-*O*-sulfonated analogues at C-3′ position (**23** and **24**) exhibited significantly stronger inhibition against isomaltase than **19**–**22**, regardless of the hydrocarbon residue length.

**Figure 3:**
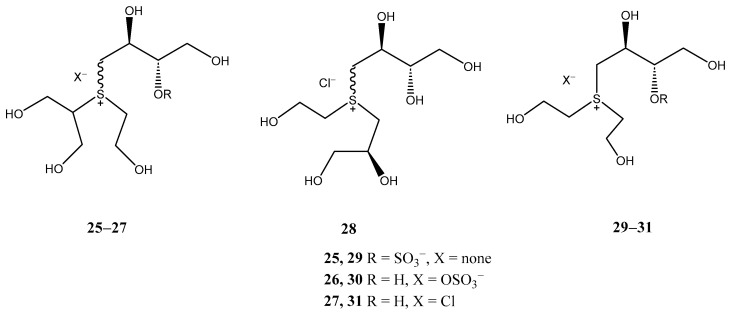
Ring-cleaved salacinol analogues.

With the aim of evaluating whether the 5-membered thiosugar is essential for the α-glucosidase inhibitory activity exhibited by sulfonium-based thiosugars, Tanabe et al. recently synthesized [23] ring-cleaved salacinol analogues (**25**–**28**) and truncated salacinol analogues (**29**–**31**) (Figure 3). This investigation was prompted by a previous report [24] demonstrating that some acyclic analogues derived from 1-deoxynojirimycin, a potent α-glucosidase inhibitor, retained activity, indicating that the five-membered ring may not be crucial for the inhibitory effect. The results for the ring-cleaved salacinol analogues revealed that these compounds were inactive, highlighting the essential role of the 5-membered thiosugar in their potent activity.

**Figure 4:**
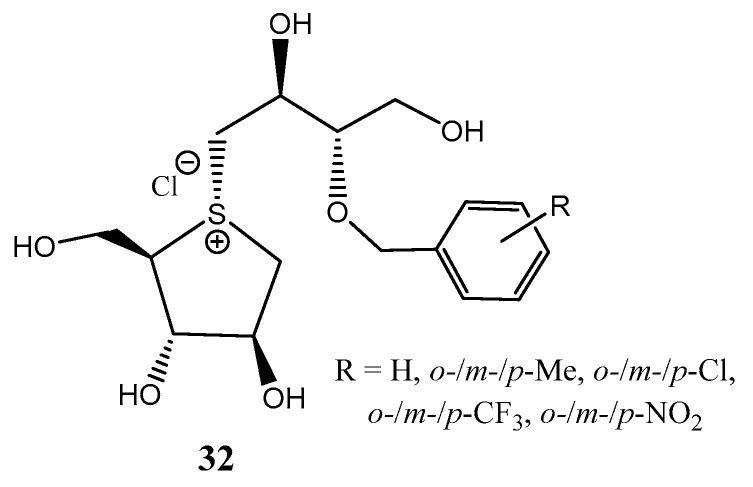
Salacinol analogues with strong α-glucosidase inhibition.

A frequent drawback found in the synthesis of sulfonium thiosugars as potential antidiabetic agents is the limited diastereoselectivity found in the alkylation reaction on the sulfur atom, hampering the scale-up of the process. Tanabe et al. reported [25] the alkylation of *O*-protected thiosugars with epoxides in hexafluoroisopropanol (HFIP), yielding a roughly 90% diastereomeric ratio (~26:1 α/β), which constitutes a 3-fold improvement compared to conventional methodologies. The excellent diastereoselectivity found was attributed to a cooperative mechanism composed of reversible *S*-alkylation and thermal isomerization. Using this methodology, derivatives **32** (Figure 4) were accessed and tested against human intestinal maltase. Those isomers with an *ortho*-substitution pattern exhibited strong inhibitory properties (IC_50_ = 0.11–0.58 µM). In vivo assays in mice revealed a high capacity for suppression of blood glucose, comparable to the antidiabetic drug voblibose [25].

### 2.2. Thioglycosides

Thioglycosides are one of the most popular glycosyl donors in glycosylation reactions, widely used in the synthesis of oligosaccharides to furnish 1,2-cis- and trans-linkages [26]. *O*-glycosides have also been obtained [27] in a stereoselective fashion starting from 1-mercapto-carbohydrates by reaction with an acid and a Cu or Co catalyst (Cu(acac)_2_, Co(acac)_2_), Ag_2_CO_3_ as the oxidant agent, and under microwave irradiation.

As aforementioned, thioglycosides exhibit a considerably higher stability towards acidic and enzymatic hydrolysis compared to natural glycosidic linkages; this feature is important for increasing their bioavailability when incorporated in bioactive compounds.

**Figure 5:**
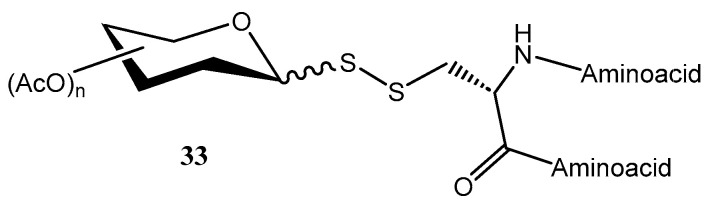
General structure of disulfide-linked glycopeptides.

For instance, Banisalman et al. prepared [28] glycopeptides **33** by conjugating mono- and disaccharides to the cysteine residues of the peptide through a disulfide linkage, involving the glycosidic position of the carbohydrate residue (Figure 5).

Incorporation of S-linked Manα1→2Man termini into oligomannose glycans (Man3, Man4) led to complete stability towards enzymatic hydrolysis mediated by *Xanthomonas manihotis* mannosidase [29]. 1-S-α-Galp(1 → 3)-β-Galp motif was incorporated [30] into oligosaccharides, leading to the isosteric unit of the epitope recognized by lytic antibodies in *T. cruzi*, the parasitic agent responsible for the Chagas disease.

An ample variety of thioglycosides have been reported as antiplasmodial [31] and antiviral agents [32], inhibitors of bacterial glycan biosynthesis [33], metabolic decoys of glycosylation [34], or heparanase inhibitors [35], among others; the latter ones are S-linked polysaccharides obtained via chemoenzymatic synthesis (heparosan synthase). Coumarin S-glycosides, obtained by thioglycoligase-mediated connection of 7-mercapto-4-methylcoumarins and *p*-nitrophenyl-d-glycopyranosides were claimed [36] to be potentially useful in bioimaging, due to their enhanced fluorescence emission properties compared to the parent coumarin.

**Figure 6:**
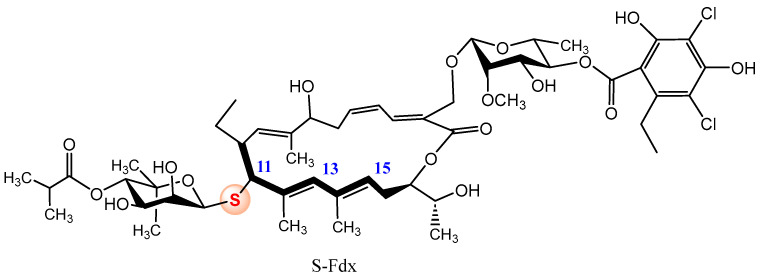
Structure of thio-Fidaxomicin.

A relevant example of the improvement of biological activity achieved by isosteric oxygen-sulfur substitution at the glycosidic position was recently reported by Gademann and coworkers [37]. The authors enhanced the acid stability of the natural glycosylated macrolactone Fidaxomicin (Fdx), approved for the *Clostridioides difficile* (C. diff.) infections; the authors replaced the *O*-glycosidic bond by the corresponding thioglycoside. Although the natural compound has shown promising activities against other pathogenic agents, its clinical use in other pathogens rather than C. diff. is hampered by its limited acid stability, making it difficult to treat, for example, stomach infections. Treatment of Fdx with Cu(ClO_4_)·6H_2_O as catalyst and the corresponding *O*-protected-1-mercapto carbohydrate, furnished a mixture of three regioisomeric glycosylated products (modest yields) at C-11 (<5%), C-13 (10%) and C-15 (24%) positions, with conserved β-configuration. Figure 6 displays the structure of the thio-isoster of Fdx, obtained as a minor compound. Degradation studies in methanolic HCl revealed that, whereas Fdx underwent degradation (t_1/2_ = 62.7 min), S-Fdx was stable over a period of 540 min [37]. Additionally, although the substitution slightly reduced the antibacterial activity, S-Fdx retained potent efficacy against C. diff. (Minimum inhibitory concentrations (MIC) ranges of 0.12–4 µg/mL) and Clostridium perfringens (MIC ranges of 0.06–0.5 µg/mL). Regioisomeric thioglycosides at C-13 and C-15 positions demonstrated to be inactive.

**Scheme 5:**
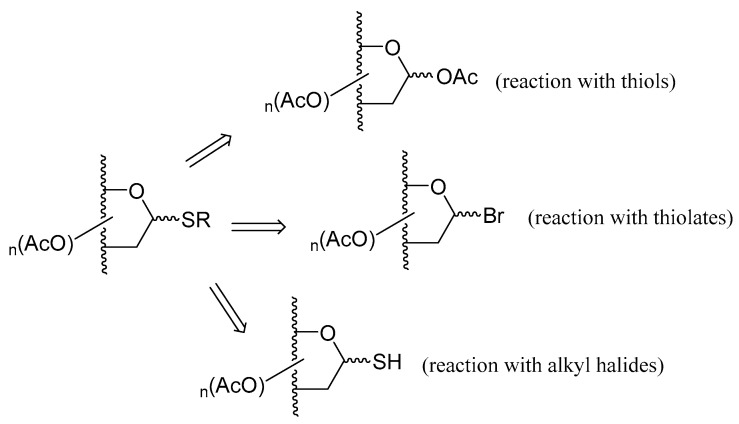
Classical procedures for preparing thioglycosides.

Classical synthetic methodologies reported for the preparation of thioglycosides include reaction of thiols with per-*O*-acetylated carbohydrates, using Lewis acids as catalysts, reaction of thiolates with acetohalosugars, or 1-thioglycoderivatives with alkyl halides. The building blocks involved in such transformations are depicted in Scheme 5.

**Scheme 6:**
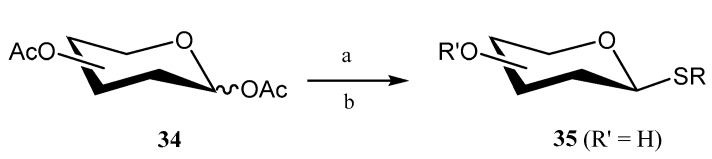
One-pot process for the thioglycoside formation and de-*O*-acetylation. Reagents and conditions: (a) PTA, HSR, DCM, MW, 30 min, 40 °C.; (b) PTA, MeOH, 90 °C, 4 h.

In the search for eco-friendly methods to synthesize thioglycosides, Luo and coworkers developed [38] an innovative methodology based on the use of phosphotungstic acid (PTA) as a catalyst for the reaction of per-*O*-acetylated saccharides **34** (d-galactose, d-xylose, l-fucose) and thiols under microwave-assisted conditions (Scheme 6). Notably, PTA proved to be highly reusable, maintaining yields of 84–88% across multiple reactions with a recovery efficiency exceeding 80%. Furthermore, methanolic PTA was successfully utilized for the one-pot de-*O*-acetylation of acetoxy compounds to give fully unprotected thioglycosides **35**.

**Scheme 7:**
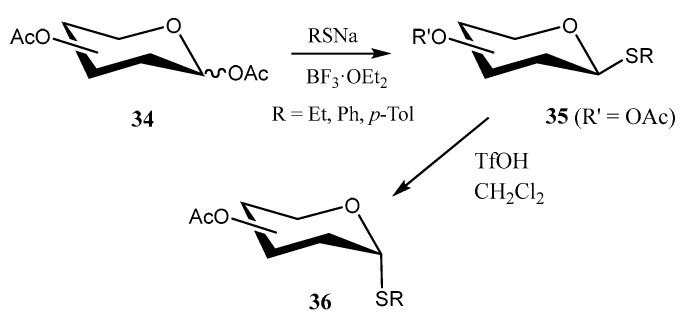
Synthesis of thioalkylglycosides.

In order to avoid handling alkyl thiols, which are frequently malodorous and toxic, Dong and coworkers developed [39] an efficient and environmentally friendly protocol that consisted of the reaction of sodium alkanethiolates with per-*O*-acetylated carbohydrates **34** in the presence of BF_3_·OEt_2_ under solventless conditions (Scheme 7). Interestingly, 1,2-trans-thioglycosides **35** could be isomerized into challenging 1,2-cis species **36** by treatment with TfOH in non-polar solvents and under mild conditions (Scheme 7).

**Scheme 8:**
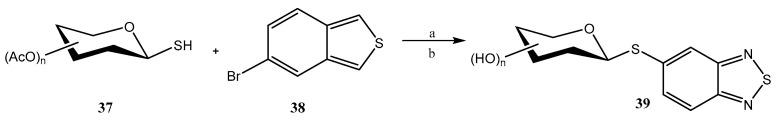
Pd-catalyzed C–S cross-coupling reaction between thiosugars and BTD. Reagents and conditions: (a) Pd catalyst 4-5 mol%, Et_3_N, 1,4-dioxane, 90 °C; (b) K_2_CO_3_ (cat.), MeOH.

Pd-catalyzed cross-coupling reactions have also been used for accessing thioglycosides. In this context, Domingues et al. employed [40] the third-generation Buchwald precatalyst (Pd-G3 Xantphos palladacycle) for functionalizing benzo-2,1,3-thiadiazole (BTD) at the C-5 position with 1-mercapto sugars **37** to give **39** (Scheme 8); the interest in such heterocyclic motif lies in the fact that it can be used as a fluorescent marker. The scope of the reaction was analyzed on pyranoses (d-Glc, d-Man, d-Gal, d-Xyl, l-Ara), furanoses (d-Rib) and disaccharides (maltose, lactose), and was found to proceed under mild conditions and to be compatible with an ample variety of functional groups. The yields were from good to almost quantitative, except for per-*O*-acetylated d-mannopyranose and d-ribofuranose, whose thioglycosides were obtained in modest yields; retention of the β-configuration was observed. The lowest yields could be significantly increased by changing the catalyst to Pd G3-MorDalphos. Products were deprotected using Zemplen-type conditions.

**Scheme 9:**
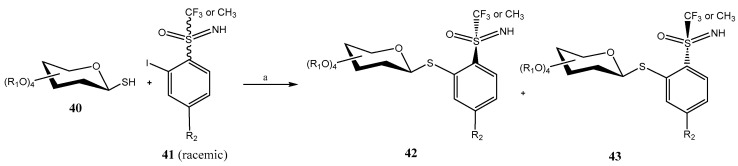
Migita cross-coupling reaction for the synthesis of thioglycosides. Reagents and conditions: (a) PdG3-XantPhos, Et_3_N (1.5 equiv), dioxane, 15–30 min.

Pd-catalyzed Migita cross-coupling reaction between a racemic mixture of *o*-iodo *S*-trifluoromethyl-*S*-arylsulfoximines **41** and a great variety of 1-mercapto sugars (mono-, di- and trisaccharides) has been used in the preparation of thioglycosides **42**/**43** [41]. For that purpose, PdG3-XantPhos was used as the catalyst, and Et_3_N as base (Scheme 9). A single β-anomer was obtained, in a 1:1 diastereomeric mixture, that was efficiently separated by either crystallization or HPLC.

**Scheme 10:**
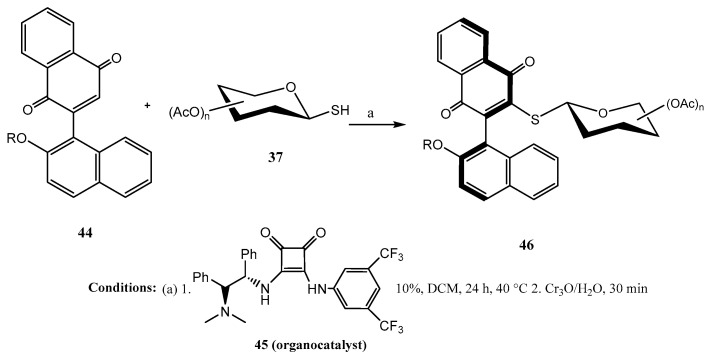
Stereoselective synthesis of naphthoquinone thioglycosides using a squaramide-based organocatalyst.

*O*-Protected 1-thio-mono- and disaccharides **37** were efficiently coupled [42] with aryl-naphthoquinones **44** (Scheme 10) in the presence of **45**, a chiral squaramide as the organocatalyst. The corresponding axially chiral thioglycosides **46** were obtained, in general, with high diastereoselectivity (14:1 to >19:1 dr). It was claimed that the hydrogen bonding established between the bifunctional organocatalyst and the quinone had a pivotal role in the activation of the substrate and the stereocontrol, locking a major conformation of the biphenyl residue at the transition state with the lowest sterical hindrance.

**Scheme 11:**
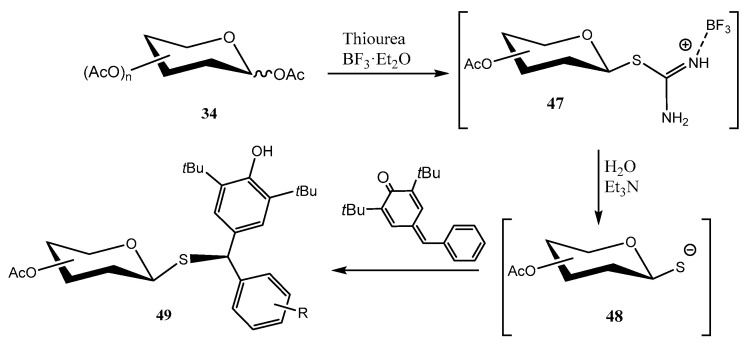
Synthesis of diarylmethyl thioglycosides.

Isothiouronium salts are interesting synthetic intermediates, and can be obtained from fully *O*-acetylated carbohydrates **34** by treatment with BF_3_·OEt_2_ to give tentatively a 1,2-acyloxonium ion [43]. Reaction of the latter with thiourea as a nucleophile, affords the corresponding isothiouronium derivative **47**, exclusively with the 1,2-trans-arrangement (Scheme 11). Hydrolysis of **47** under weakly basic conditions (Et_3_N) gives access to a transient thiolate (**48**), which exerts a 1,6 nucleophilic addition on *p*-quinone methide, with the subsequent formation of diarylmethyl thioglycosides **49**.

**Scheme 12:**
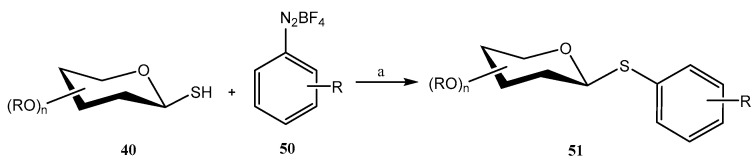
Synthesis *S*-aryl thioglycosides. Reagents and conditions: (a) CuCl (5%), DBU (1 eq), MeCN, 0–5 °C.

Aryldiazonium salts are known for their inherent electrophilicity, and have been used in the formation of C–S bonds. Building on this property, Venkatesh et al. developed [44] a reaction between thiosugars **40** and activated aryldiazonium salts **50** (Scheme 12) to efficiently form C–S bonds (*S*-arylation, compounds **51**). The optimized conditions involved the use of CuCl as the catalyst, DBU as the base, low temperatures (0–5 °C) and short reaction times (<5 min). Under these conditions, diazosulfide (R-S-N=N-Ar) is not observed, and only small amounts of the competing dimer (R-S-S-R) was obtained. The reaction was compatible with an ample number of protecting groups (NHAc, Ac, Bn, Bz), and even with unprotected carbohydrates; however, in this case, the ratio of the undesired dimeric disulfides increased [44]. It was postulated that the key intermediate is a free radical, obtained upon reaction of the aryldiazonium salt with the Cu(I) catalyst. This mechanism was demonstrated by the addition of a free radical scavenger (TEMPO), furnishing only traces of **51** [44].

**Figure 7:**
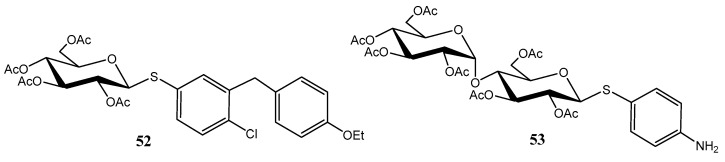
Thio-analogues of dapagliflozin and arbutin.

This approach enabled the successful synthesis of thio-analogues of bioactive compounds, including the antidiabetic agent dapagliflozin (**52**) [45,46], and the tyrosinase inhibitor arbutin (**53**) (Figure 7).

**Scheme 13:**
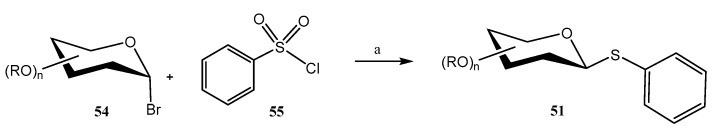
Synthesis of boron-mediated arylthioglycosides. Reagents and conditions: (a) B_2_pin_2_ (2.5 equiv), PPh_3_ (3.0 equiv), KOH (4.0 equiv), DME, 25 °C.

A stereospecific metal-free synthesis of aryl thioglycosides **51** was achieved by using a boron catalyst for promoting a reductive deoxygenation coupling reaction between *O*-protected α-acetobromohexoses **54** (also di- and trisaccharides) and sulfonyl chlorides **55** (Scheme 13) [47]; B_2_pin_2_ (Bis(pinacolato)diboron) and PPh_3_ were used as additives. This afforded exclusive β-stereoselectivity, even with challenging β-mannosides and β-rhamnosides. This methodology also enabled the preparation of thioglycoconjugates of bioactive and natural compounds including naproxen, ibuprofen, indomethacin and lithocholic acid.

**Scheme 14:**
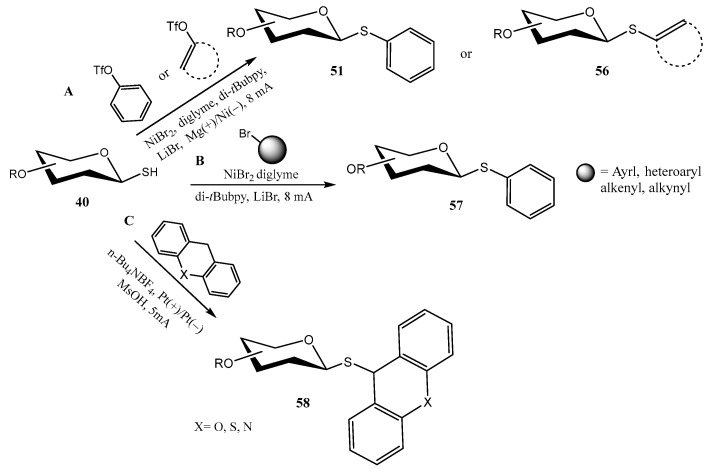
Electrochemical synthesis of thioglycosides.

Synthetic organic electrosynthesis involving cross-coupling reactions has emerged as a valuable strategy [48], particularly useful for forming new C–C, C–O, C–N, and C–S bonds. In connection to the preparation of thioglycosides, *O*-protected 1-thiosugars **40** were coupled to preactivated phenols and ketones (aryl/alkenyl triflates) (Scheme 14A) [49], aryl/alkenyl/alkynyl bromides (Scheme 14B) [50], or xanthenes/heteroxanthenes (Scheme 14C) [51] to give the corresponding β-configured thioglycosides **51**, **56**, **57**, **58**. Reactions A and B from Scheme 14 were catalyzed by Ni (NiBr_2_·diglyme), requiring a ligand (di-*t*Bubpy) and an additive (LiBr), and were performed in undivided cells. In a plausible mechanism, some of the key steps would be the cathodic reduction of the Ni salt to Ni(0), the oxidative addition into the triflates/bromides, and the single electron oxidation of **40** to give a thiyl free radical [49].

On the contrary, reaction C did not require a metallic catalyst; n-Bu_4_NBF_4_ was used as the electrolyte, and MsOH as an additive. Control experiments and cyclic voltammetry assays suggest that both the thiol group and the xanthene underwent an anodic oxidation, to give free radicals (the latter at the benzylic position); cross-coupling reaction between both free radical species would furnish thioglycosides **58** [51]. Reduced current intensity compared to pathways A and B (5 mA vs. 8 mA, Scheme 14) proved to be essential, due to the instability of **58** at higher currents.

**Scheme 15:**
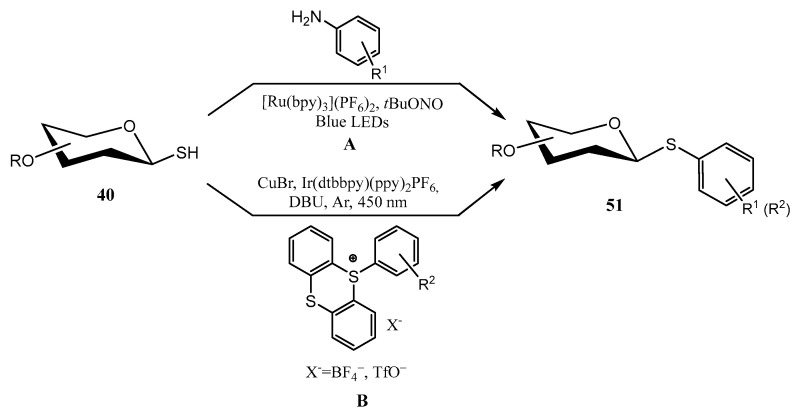
Photoredox reactions for the synthesis of thioglycosides.

Photoredox processes have also emerged as a valuable alternative to classical S-glycosilation reactions, as they can be conducted under mild conditions, and at late synthetic stages, due to their compatibility with an ample variety of functional groups. In this context, the contributions of Messaoudi’s [52] and Ji’s [53] groups are remarkable. *O*-Protected sugar-derived thiols **40** could be transformed into aryl thioglycosides **51** (Scheme 15A) using reactive aryl radicals obtained from in situ generated diazonium salts (anilines + tBuONO) upon irradiation with visible light (blue LEDs) in the presence of a photoredox catalyst ([Ru(bpy)_3_](PF6]_2_) [52], through a Stadler-Ziegler reaction. Diazonium salt is hypothesized to react with sugar-derived thiol to give a photolabile diazosulfide (Sugar-S-N=N-Ar). Next, a single electron transfer (SET) process takes place from the excited state of the photocatalyst to the diazosulfide, which releases N_2_, a thiolate and a reactive aryl free radical. The combination of these reactive species furnishes a thioether radical anion, which through a second SET process gives the thioglycoside and restores the Ru-based catalytic cycle [52].

The photoredox formation of thioglycosides using thianthrenium salts (TT) as aryl donors and 1-thiosugars **40** has also been explored [53] (Scheme 15B). Optimization of the reaction conditions indicated that the use of Ir(dtbbpy)(ppy)_2_PF_6_ as the photoredox catalyst, CuBr as co-catalyst, DBU as base and irradiation under blue light (450 nm) achieved the best results. A wide scope was also found, as it was compatible with numerous electron-donating/withdrawing substituents, mono-and disaccharides, and proved to be efficient also for the derivatization of natural products/drugs. These include derivatives of estrone, gemfibrozil, pyriproxyfen, or flurbiprofen [53].

**Scheme 16:**
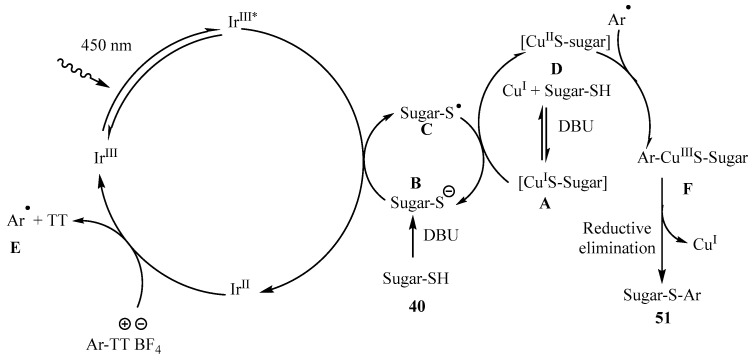
Plausible mechanism for the photoredox preparation of thioglycosides **56**.

**Scheme 17:**
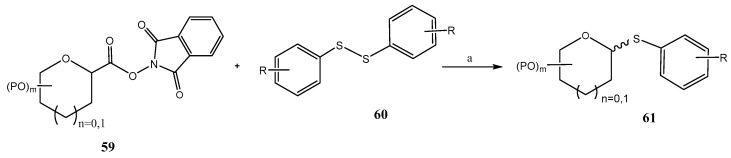
Synthesis of thioglycosides from glycosyl redox-active species. Reagents and conditions: (a) DIPEA, DMSO, 12 W LEDs, r.t.

The proposed mechanism for the photoredox formation of thioglycosides **51** from TT is depicted in Scheme 16 [53]. Control experiments revealed that, analogously to what was found to the preparation of thiolglycosides from aryldiazonium salts (Scheme 15, pathway A), aryl and thiyl free radical species (E and C) were involved. Thiolate anion was postulated to quench the excited state of the photocatalyst, to give Ir^II^ and the thiyl radical. Subsequently, upon a SET process between the quenched catalyst and the thianthrenium salt, the aryl radical intermediate E was generated. Additionally, the Cu^I^-thiol complex A was oxidized by **C** to give the corresponding Cu^II^ complex D. Thioglycoside 51 was then obtained after oxidative ligation between the aryl radical E and D, followed by reductive elimination (Scheme 17) [53].

Another recent application of photoredox processes in the preparation of thioglycosides was recently reported by Zhang et al. (Scheme 17) [54]. In this approach, that does not require metal photocatalysts, glycosyl redox-active esters **59** and disulfides **60** were irradiated with a blue LED (455 nm) in the presence of DIPEA to give thioglycosides **61** with acceptable stereoselectivity. Instead of a SET process, commonly proposed when metal photocatalysts are present (e.g., Scheme 16), a photoactive electron-donor-acceptor (EDA) complex between **59** and DIPEA was proposed as the key transient species.

**Figure 8:**
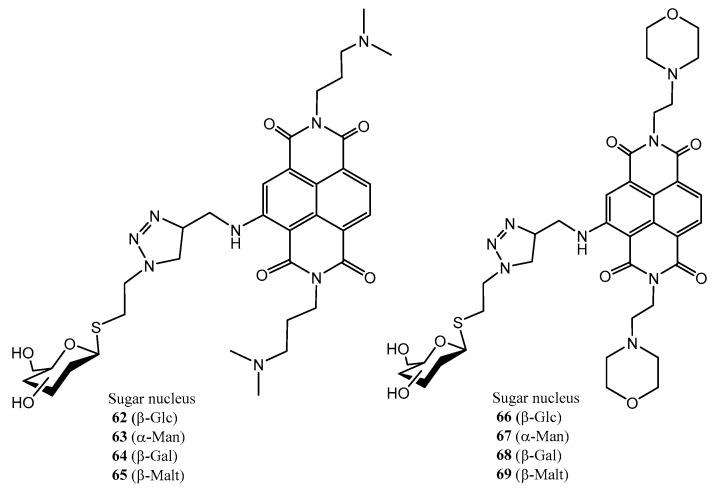
Thiosugar-NDI conjugates as G-quadruplex ligands.

**Figure 9:**
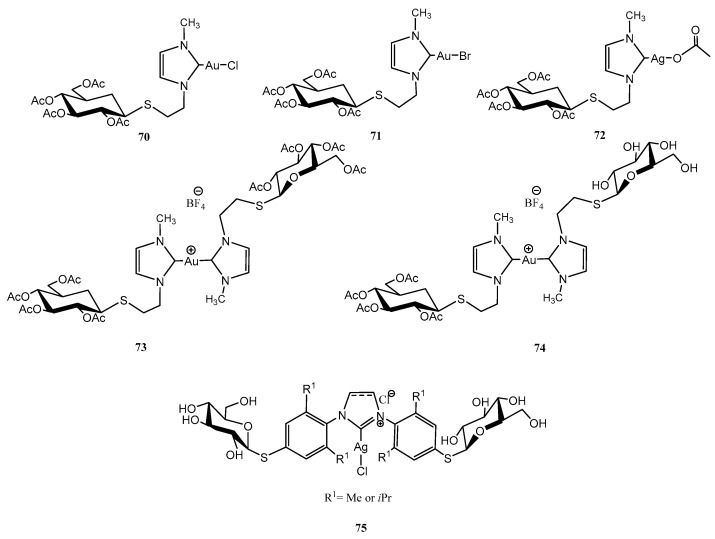
Bioactive NHCs containing thioglycosides.

**Figure 10:**
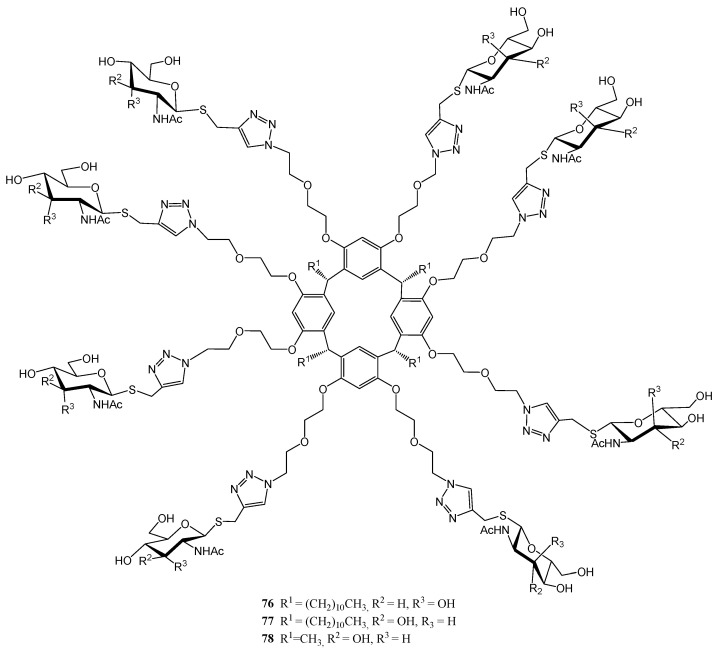
Structure of multivalent β-thio-glycoclusters.

Figure 8, Figure 9 and Figure 10 depict three families of thioglycosides endowed with relevant bioactivities: naphthalene diimide (NDI) conjugates (**62**–**69**) [55], *N*-heterocyclic carbenes (NHCs) [56,57] (**70**–**75**), and multivalent β-thio-glycoclusters (**76**–**78**) [58].

Naphthalene diimide derivatives (NDI) were designed as ligands of the G-quadruplex, a guanine tetrad located in key regions of the genome; binding to G-quadruplex can confer anticancer and antiparasitic properties. The carbohydrate residue may be recognized by the glucose transporters (GLUT), overexpressed in cancer cells, whereas the thioglycosides-type linkage can provide enhanced hydrolytic and enzymatic stability [55]. Notably, dimethylamino derivatives **62**–**65** exhibited improved cytotoxicity against both, colon cancer cells and parasites (*T. brucei* and *L. major*), compared to morpholine-containing counterparts **66**–**69** (Figure 8); this was attributed to a less efficient binding to G-quadruplexes of the latter compounds. Thioglycosides, although exhibiting a similar potency against the cancer cell compared to their bioisosteric *O-*glycosides, showed a better selectivity against non-tumour cells. Thiomaltosyl derivative **65** was found to be the best compound (submicromolar activity against colon cancer cell, selectivity index = 9.8). This compound also had nanomolar activity against *T. brucei* [55].

NHCs, monodentate ligands that act as two-electron donors, are analogous to phosphines, although with stronger donation capacity; structural modifications can tune their stereoelectronic properties [59]. Numerous Ag/Au-NHC complexes have shown anticancer properties, like those depicted in Figure 9. Ag-NHC complexes **70**–**74**, although less stable in aqueous solutions than Au-counterparts, exhibited superior cytotoxicity against ovarian A2780 cancer cells (IC_50_ = 1.1–1.3 μM, similar to the chemotherapeutic agent cisplatin, IC_50_ = 1.5 μM) [56]. Au-NHC complexes demonstrated strong binding properties to thiol-rich biomolecules, acting as potent inhibitors of thioredoxin reductase (TrxR).

A Pd-catalysed Migita cross-coupling reaction and a mechano-chemistry approach were used for the preparation of Ag-NHC thioglycosides **75** (imidazolium and imidazolinium) [57]. Imidazolinium derivative with R^1^ = Me exhibited the best antiproliferative activity against HCT116 tumour cells (colon, 41% viability at 10 µM concentration).

Cristófalo et al. reported [58] the preparation of calix[4]resorcinarene-based octavalent ligands **76**–**78** (Figure 10), bearing β-S-GlcNAc (*N*-acetylglucosamine) and, for the first time, β-S-AllNAc (*N*-acetylallosamine) residues as recognition elements for the lectin Wheat Germ Agglutinin (WGA). These compounds were obtained via copper(I)-catalyzed azide–alkyne cycloaddition (CuAAC) reaction between the corresponding alkynyl thioglycosides and the octa-azido scaffold derived from resorcinarene. Turbidimetry and isothermal titration calorimetry assays revealed that the highest affinity was found for allosamidine-derivative **76** [58], a hitherto unknown property for such monosaccharide. This behavior was explained through in silico calculations.

#### Thioglycosides in Pseudo-Disaccharides and Pseudo-Oligosaccharides

The thioglycosidic linkage has also been employed to connect a second sugar unit through different positions, and thus, to obtain pseudo-disaccharides and pseudo-oligosaccharides.

**Scheme 18:**
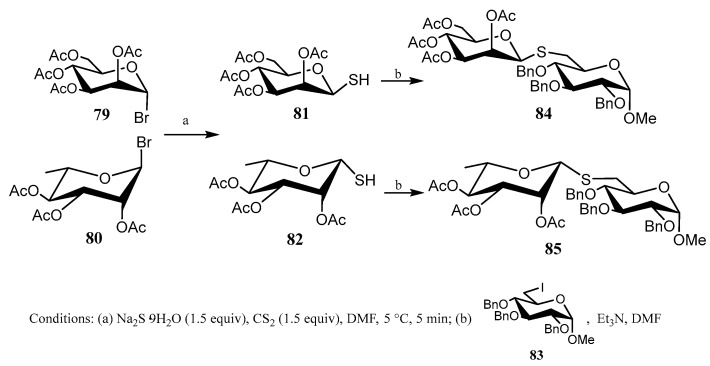
Synthesis of pseudo-disaccharides from 1-thio-β-d-mannosides and 1-thio-β-l-rhamnosides.

In this context, Misra’s group developed [60] a simple two-step methodology for preparing pseudo-disaccharides connected through a thioglycosidic linkage. The approach involves treating per-*O*-acetyl-α-d-mannopyranosyl bromide (**79**) or per-*O*-acetyl-β-l-rhamnopyranosyl bromide (**80**) with a mixture of Na_2_S·9H_2_O and CS_2_ for a short time (5 min); 1-mercapto derivatives **81** and **82** were obtained, respectively. Subsequent addition of an electrophilic carbohydrate, like 6-iodo-glucopyranoside **83**, and Et_3_N gave β(1→6) pseudo-disaccharides in good yield (Scheme 18).

**Scheme 19:**
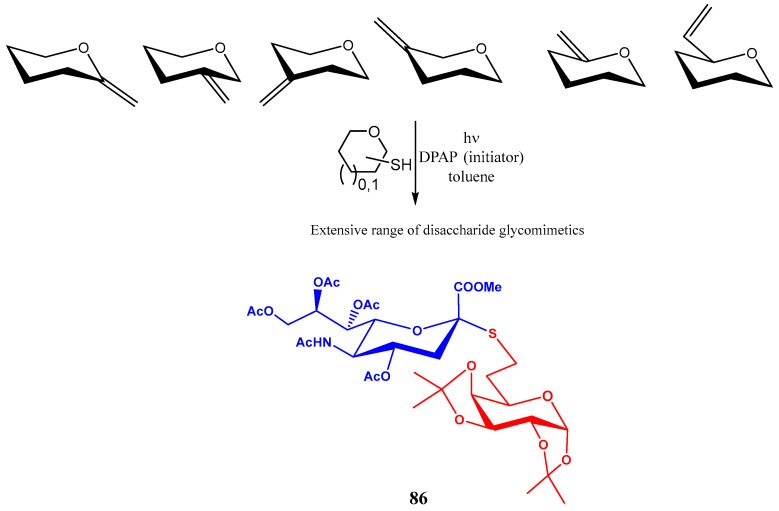
Reactions of enopyranoses bearing an exocyclic double bond.

Photoinitiated thiol-ene coupling reactions were used by Borbás and co-workers for preparing *S*-linked pseudo-disaccharides [61]. These glycomimetics were obtained by UV-induction of hydrothiolation reactions between unsaturated sugars bearing an exocyclic double bond at C1, C2, C3, C4, C5 and C6 positions and a thiol-containing sugar (Scheme 19). 2,2-Dimethoxy-2-phenylacetophenone (DPAP) was used as the photoinitiator. Reaction proceeds in a two-step pathway: reversible addition of the thiyl free radical, and the irreversible donation of hydrogen atom from a thiol; although the stereoselectivity strongly depends on the carbohydrate involved, a preferential axial H-transfer is frequently observed, with the thiol substituent occupying the equatorial position [61].

**Scheme 20:**
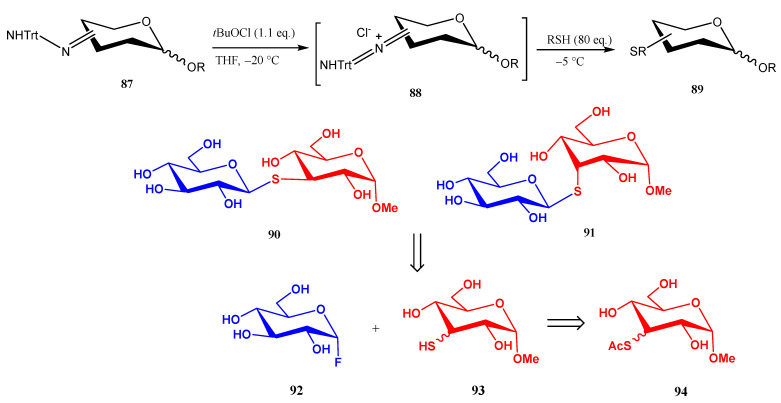
Synthesis of thiosugars from sugar-derived trityl hydrazones.

Using this methodology, the sialyl thioglycoside **86** was obtained in good yield (Scheme 20).

A straightforward alternative for preparing *O*-unprotected *S*-linked pseudo-disaccharides consists of the preparation of sugar-derived trityl hydrazones **87**, which upon treatment with *t*BuOCl generate a chloro-azo intermediate on the sugar moiety [62]. Thermolysis of such intermediate in the presence of an excess of simple thiols generates thioethers **89** in up to almost quantitative yield (Scheme 20).

This methodology was applied to the preparation of pseudo-disaccharides **90** and **91**, which were obtained by treatment of trityl hydrazone **87** with AcSH to give **94**, followed by hydrolysis of the acetyl moiety, and coupling of the corresponding 3-thiols **93** with glucopyranosyl fluoride **92** (Scheme 20) [62].

**Scheme 21:**
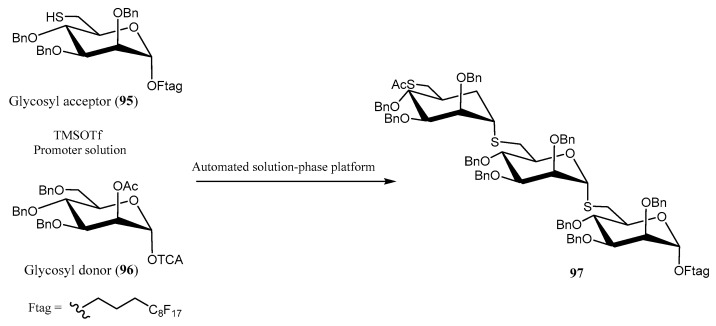
Automated approach for the synthesis of oligomannopyranosides.

Automated syntheses have also been applied to the selective incorporation of *S*-glycosides into oligomannopyranosides [63] using an inverse glycosylation protocol instead of the conventional methodology. The synthesis of trisaccharide **97** was accomplished with good yield (73%) employing reactive glycosyl acceptor **95**, glycosyl donor **96**, and TMSOTf as the promoter (Scheme 21). The process comprised three sequential cycles of glycosylation coupling, deprotection, and purification. The automated protocol was executed on a platform equipped with a robotic arm and dual syringe pumps, enabling precise delivery of reagents to an array of double-jacketed reaction vessels. The reaction setup was maintained under an inert atmosphere, ensuring controlled and reproducible conditions for each step of the synthesis [63].

### 2.3. 3-Thiosugars

Incorporation of a sulfur atom at C-3 position of a sugar residue has been used for accessing valuable synthetic intermediates, as well as for preparing derivatives with potential biological activities [64]. Despite that, this kind of sulfur-containing carbohydrates is significantly less studied compared to other positions on the sugar, like the endocyclic oxygen, or the glycosidic position. A frequent approach for accessing 3-thiosugars is the reaction of glycals with different thiols and catalysts, as displayed in this section.

**Scheme 22:**
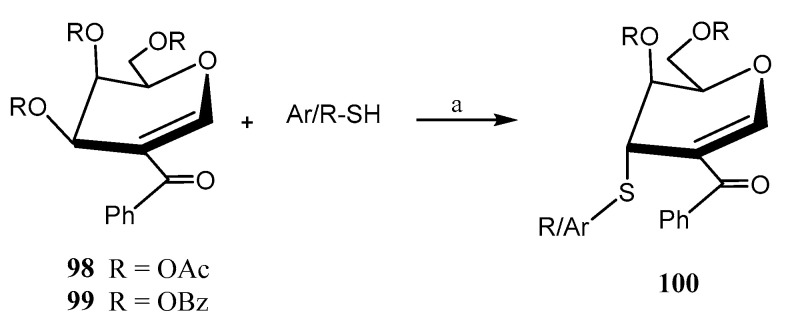
Synthesis of a library of 3-aryl/alkyl thiosugars prepared from 2-ketoglycals. Reagents and conditions: (a) Et_3_N (1 equiv.), MeCN, r.t.

Mukherjee et al. reported [65] the biomimetic synthesis of a family of 3-thiosugars (**100**, Scheme 22) via reaction of 2-ketophenyl-glycals **98**, **99** (d-glucal, d-galactal, l-rhamnal) with different thiols. The regio- and stereoselective displacement of the acetoxy group at the C-3 position of the glycal was achieved using aromatic and cyclic aliphatic thiols under mild basic conditions (Et_3_N), furnishing excellent axial selectivity. The presence of a carbonyl group at C-2 was found to be crucial [65]. It was hypothesized that the thiol attacks the carbonyl group at C-2, and then it undergoes a 1,3-migration through the opposite face of the acetoxy group at C-3. This process was inspired by the thiolation of glucosamine by cytosolic esterases.

**Scheme 23:**
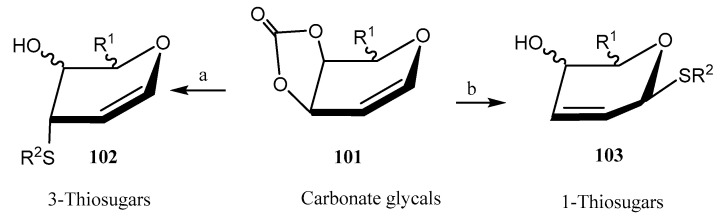
Synthesis of 1- and 3-thiosugars using Co and Pd-based catalysts. Reagents and conditions: (a) RSH, Co(BF_4_)_2_ catalyst, CH_2_Cl_2_, 35 °C; (b) RSH, Pd_2_(dba)_3_ catalyst, 100 °C.

Liu and coworkers reported [66] the stereo- and regioselective synthesis of thiosugars by reaction of 3,4-*O*-carbonate glycals **101** with different thiols; competitive experiments revealed that the thiol group reacted with total chemoselectivity in the presence of other nucleophilic moieties, like alcohols, phenols, amides, or amines. A regiodivergent approach was developed, as depending on the catalyst employed, either 3-thiosugars (with Co(BF_4_)_2_, axial position, compound **102**) or 1-thiosugars (with Pd_2_(dba)_3_, equatorial position, compound **103**) were obtained, with general good yields (Scheme 23). Based on computational calculations, it was hypothesized that under Pd catalysis, the thiol group established hydrogen bonding on the top face with the oxygen atom at C-4 position. Nevertheless, coordination with Co took place through the bottom face.

**Scheme 24:**
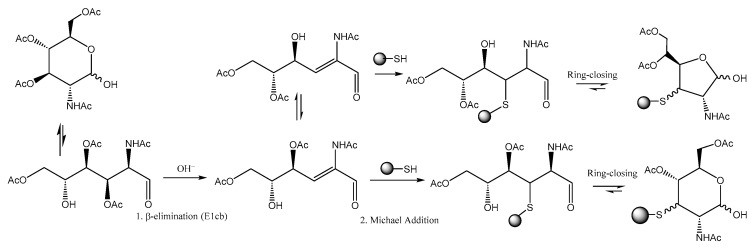
Proposed two-step elimination–addition mechanism for S-glyco-modification.

Chen and co-workers noticed [67] that per-*O*-acetylated unnatural monosaccharides and the cysteine residues on proteins undergo an atypical glycosylation process, yielding 3-thiolated sugars in their hemiacetal form. In their study, the authors elucidated an elimination-addition mechanism, which involves a base-promoted β-elimination step followed by a Michael addition of the cysteine residue to the α,β-unsaturated aldehyde. (Scheme 24). This process, called *S*-glycosylation, might compromise the specificity of the metabolic glycan labelling (MGL) used, for example, in glycan tagging with fluorophores.

### 2.4. Other Thiosugars

**Scheme 25:**
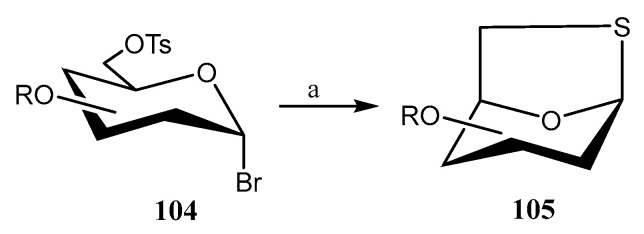
Synthesis of 6-anhydro-1-thio-β-d-hexopyranose derivatives. Reagents and conditions: (a) Na_2_S·9H_2_O (2 equiv), CS_2_ (2 equiv), DMF, r.t.

1,6-Anhydro-1-thio-β-d-hexopyranose derivatives are relevant molecules in Medicinal and Synthetic Chemistry, functioning as precursors of glycomimetics. Misra’s group has developed [68] a fast, effective, and scalable method for their synthesis by treatment of protected 6-*O*-tosylated glycopyranosyl bromide derivatives **104** with Na_2_S·9H_2_O (2 equiv.) and CS_2_ (2 equiv.) at rt (Scheme 25). The reaction was completed in just 5 min, yielding the corresponding thiolevoglucosan derivatives **105** with yields ranging from 82% to 92%.

**Scheme 26:**
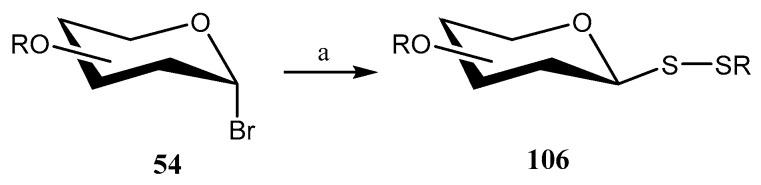
Synthesis of unsymmetrical glycosyl disulfides. Reagents and conditions: (a) CS_2_ (2 equiv.), Na_2_S·9H_2_O (2 equiv.), RSSR, DMF, 0 °C, rt, 5–20 min.

Using the same combination of reactants, the authors extended [69] the methodology to synthesize unsymmetrical glycosyl disulfides directly from glycosyl bromides by incorporating symmetrical disulfides. Under optimized conditions, a series of anomeric glycosyl bromides **54** were treated with symmetrical alkyl, aryl, and glycosyl disulfides, affording unsymmetrical β-glycosyl disulfides **106** in yields ranging from 72% to 90% (Scheme 26).

## 3. Se-Containing Carbohydrates

The incorporation of selenium into organic frameworks has led to the development of potent bioactive compounds with antioxidant [70], antiviral [71], antiparasitic [72], anti-Alzheimer’s [73,74,75], or anticancer properties [76,77,78,79,80], among others. Combining the diverse biological activities of selenium with the unique structural features of carbohydrates offers a promising strategy for the design of novel drug candidates with enhanced therapeutic potential.

### 3.1. 4′- and 5′-Selenosugars

**Figure 11:**
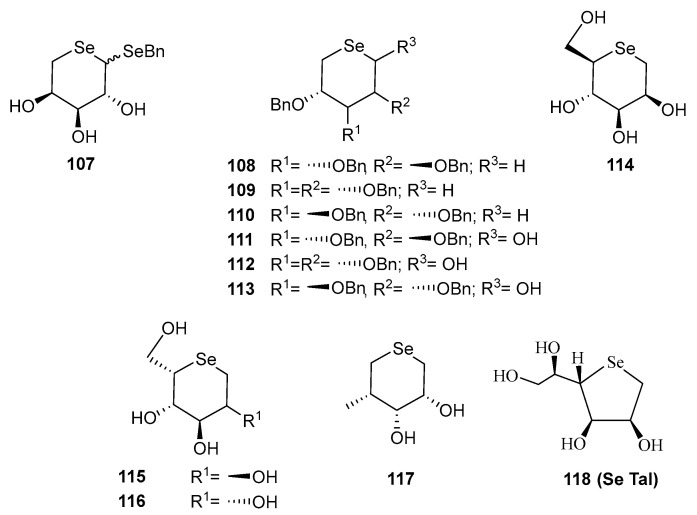
Structures of some of representative examples of selenosugars.

Interest in replacing the endocyclic oxygen atom in carbohydrates by selenium dates back to the 1970’s. However, most early attempts were unsuccessful, either yielding undesired by-products, or producing the desired selenosugars in very low yields (e.g., derivative **107** (Figure 11) [81]. Later, Schiesser and co-workers, in the search for water-soluble antioxidants, pioneered [82] a more practical approach for synthesizing selenosugars (**108**–**110**). This was achieved through the thermolysis of per-*O*-bezylated-5-benzylseleno formates of d-ribo-, xylo- and arabino-configurations. Their method involved an intramolecular attack of the benzylseleno scaffold, and elimination of CO_2_ and phenylselenoate.

To prepare reducing selenosugars, the same group reported [82] the SmI_2_-mediated transformation of *O*-protected 5-benzylseleno aldoses into derivatives **111**–**113** via an intramolecular homolytic substitution [83]. Nevertheless, only d-arabino-configured **113** was obtained in pure form and with moderate yield. Unfortunately, none of such compounds could be successfully deprotected.

**Scheme 27:**
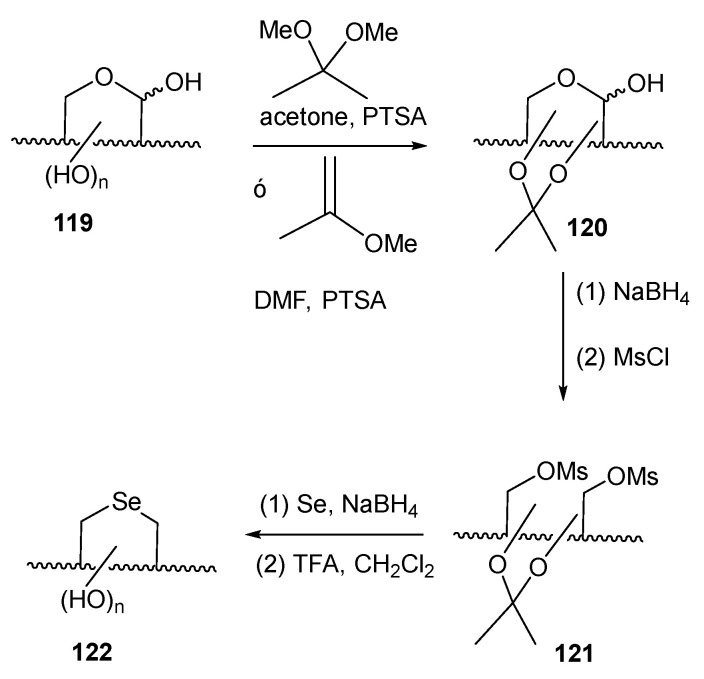
General procedure for the preparation of unprotected selenosugars.

Liu and Pinto developed [84] a procedure for achieving unprotected selenofuranoses and pyranoses by using acetals as protecting groups in their efforts to synthesize selenonium sulfates as analogues of natural salacinol and kotalanol (Figure 2), potent α-glucosidase inhibitors. Their strategy for accessing the selenosugars was based on four steps: appropriate *O*-protection of the starting carbohydrate with isopropylidene groups, reduction of the latent aldehyde of the reducing sugar, di-*O*-mesylation and double nucleophilic displacement with in situ generated Na_2_Se (treatment of elemental selenium with the appropriate number of equivalents of NaBH_4_), as depicted in Scheme 27 [84]. This simple synthetic pathway inspired Schiesser’s and other groups to access a wide variety of pyranoses and furanoses with diverse configurations. These included compounds like **114**–**116**, **118** [83,85], which are efficient scavengers of hypohalous acids and **117**, a good mimetic of glutathione peroxidase (GPx) [86].

Undoubtedly, due to its synthetic accessibility, the most extensively studied compound in this series is 1,4-anhydro-4-seleno-d-talitol (**SeTal**, **118**, Figure 11). It has been considered as a privileged structure because of the diverse biological properties it exhibits. This water-soluble compound has demonstrated [87] potent scavenging activity against oxidizing agents, like HOCl and HOBr, the former being produced by the enzyme myeloperoxidase (MPO) to eliminate pathogens from inflamed tissues. Alongside other analogues with different configurations, **SeTal** and its derivatives have shown antioxidant potency up to twice that of their sulfur-counterparts. Moreover, **118** has been shown to repair damaged skin tissues in animal models, including diabetic wounds [87]. **SeTal** exhibits remarkable stability under acidic conditions (as those found when drugs have an oral administration) and also in artificial gastric or intestinal fluids, achieving steady-state intracellular concentrations ranging 2–10 μM. However, the exact internalization mechanism remains undetermined [88]. In vitro experiments have revealed that **SeTal** protects against oxidative damage in human coronary artery cells and mouse aortic rings [88]. Interestingly, topical application of **118** to induced skin injuries in mice modulated inflammatory markers [89]. Furthermore, its incorporation into gelatin and alginate polymeric films, either alone, or in combination with hydrocortisone or vitamin C, has recently shown promise as a treatment of atopic dermatitis [90]. Studies on its potential hepatotoxicity revealed [91] that **SeTal** only affects hepatic cells viability at very high concentrations, much higher than its potential therapeutic dose, making it a promising candidate for drug development.

**Scheme 28:**
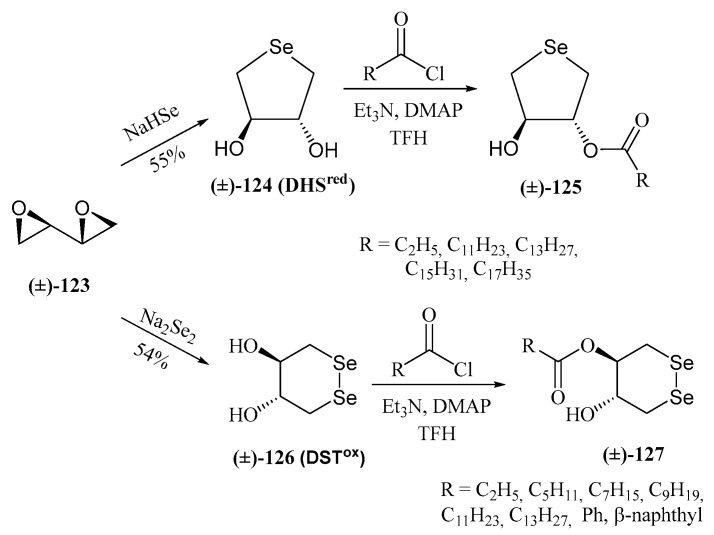
Preparation of trans-dihydroxy selenonanes and diselenanes and lipophilic derivatives.

**Scheme 29:**
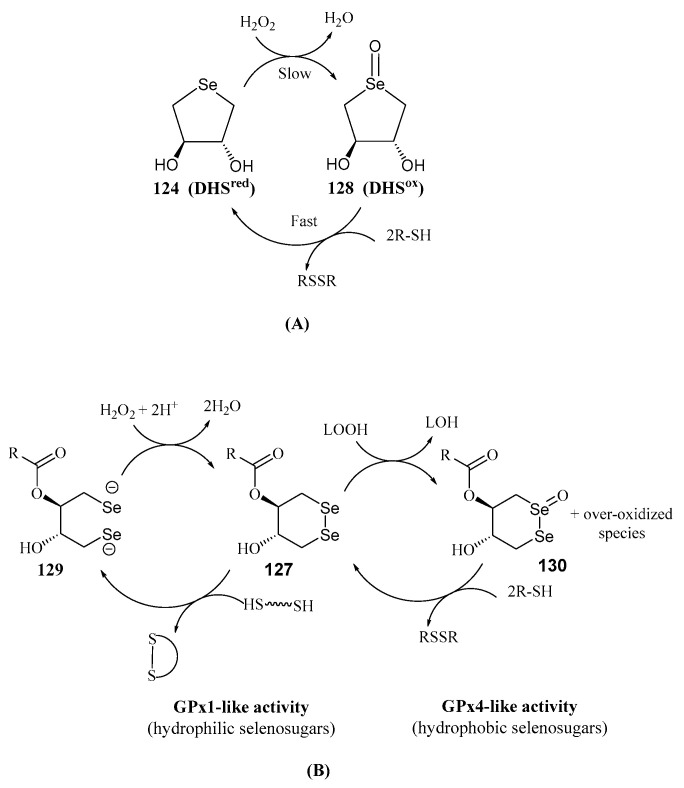
Catalytic cycles for the GPx-like activity of DHS^red^ (**A**) and DST^ox^ (**B**).

Another significant contribution to the field of water-soluble selenosugars was made by Iwaoka and Tomoda. They synthesized *trans*-dihydroxy selenonane **124** (DHS^red^) and diselenane **126** by nucleophilic opening of racemic 1,3-butadiene diepoxide with NaHSe or Na_2_Se_2_, respectively (Scheme 28) [92]. DHS^red^ was shown to mimic the catalytic cycle of GPX by reducing H_2_O_2_ in the presence of a thiol-containing compound as a cofactor such as dithiothreitol (DTT) [93]. GPx is a metalloenzyme that maintains the homeostasis in Reactive Oxygen Species (ROS) levels by eliminating H_2_O_2_ and alkyl peroxides, with glutathione (GSH) as a cofactor. The proposed catalytic cycle in aqueous media involves the slow oxidation of DHS^red^ with H_2_O_2_ to produce the selenoxide **128**, which is subsequently reduced by DTT to give DSH^red^ [93] (Scheme 29A).

DSH^red^ exhibited superior ROS scavenging capacity compared to its non-cyclic isomer. The enhanced activity was attributed to increased HOMO energy due to the strain caused by the cyclic structure [94], facilitating oxidation. Unexpectedly, when GPx-like activity was tested in MeOH, the reaction became much more complex. Over-oxidized species, like hydroxyselenonium and hydroxy perhydroxyselane were suggested to play an active role in the scavenging process [95].

DHS^red^ also demonstrated [96] radio-protective effects in mice when administrated intraperitoneally before and after the irradiation with ^60^Co. Reduced DNA damage, decreased lipid peroxidation, and down-regulation of pro-inflammatory genes were observed [96]. These effects were similar to those exhibited by the seleno-amino acid SeMet. The mechanism included GPx-dependent DNA repair enhancement [97].

Monoesterification of DHS^red^ with acid chlorides derived from propionic, lauric, myristic, palmitic, and stearic acids yielded fatty acid conjugates (**125**), with lipid peroxide (LOOH) scavenging activity in lecithin/cholesterol liposome membranes [98]. The myristate derivative displayed interfacial redox activity. Amphiphilic diselenides (**127**) were also synthesized [99].

Diselenides such as **127** (Scheme 28) were shown [100] to mimic protein disulfide isomerases (PDIs), enzymes critical for redox homeostasis in the endoplasmic reticulum and in the prevention of amyloid plaque pathogenicity. Furthermore, GPx-like activity was observed for **126** and **127**. Depending on their hydrophilic (R = H, C3) or hydrophobic (R = C6–C14) nature, GPx1- or GPx4-like activity predominated [99], respectively. These compounds reduced H_2_O_2_ or lipid peroxides (LOOH) to harmless H_2_O and an alcohol (LOH), respectively (Scheme 29B). Antioxidant properties were also confirmed in cultured cells [99]. A similar behavior was proposed for DHS^red^ (**124**) and monoesters counterparts (**125**) [101].

**Scheme 30:**
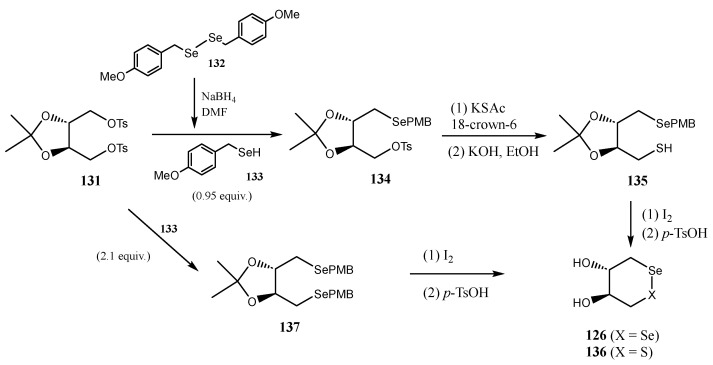
Synthesis of *trans*-dihydroxy diselenide **126** and selenenyl sulfide **136**.

Mugesh and co-workers described [102] the synthesis of enantiomerically pure trans-dihydroxy diselenide (**126**) and selenyl sulfide (**136**) as antioxidants to protect erythrocytes (RBCs, Red Blood Cells) from oxidative stress-induced eryptosis (programmed RBC death). The synthesis involved treatment of 1,4-di-*O*-tosyl-2,3-*O*-isopropylidene-l-threitol **131** with substoichiometric selenol **133** (obtained by reduction of *p*-methoxybenzyl diselenide **132**) to give benzyl selenide **134**. Subsequent nucleophilic displacement of the second tosylate with KSAc, followed by I_2_-mediated oxidation afforded *O*-protected selenyl sulfide **135** (Scheme 30). Final deprotection under acidic conditions furnished **136**. Alternatively, treatment of ditosylated **131** with an excess of selenol **133** gave the dibenzylselenide **137**. Final oxidation and deprotection led to the expected diselenide **126** [102].

When RBCs were exposed to H_2_O_2_ to simulate severe oxidative stress, treatment with **126** and **127** reduced ROS levels, demonstrating effective antioxidant activity in cells. Structural aspects proved critical; decreasing conformational flexibility (via isopropilidene protection) or replacing selenium atoms with sulfur impaired activity [102]. *O*-protected derivatives exhibited high toxicity, even without H_2_O_2_. Inhibition of glutathione reductase (GR), leading to reduced glutathione (GSH) levels, significantly diminished the protective effects of **126** and **136**. This confirmed that their antioxidant activity primarily relies on GPx-like mechanisms, with GSH as a cofactor [102].

**Scheme 31:**
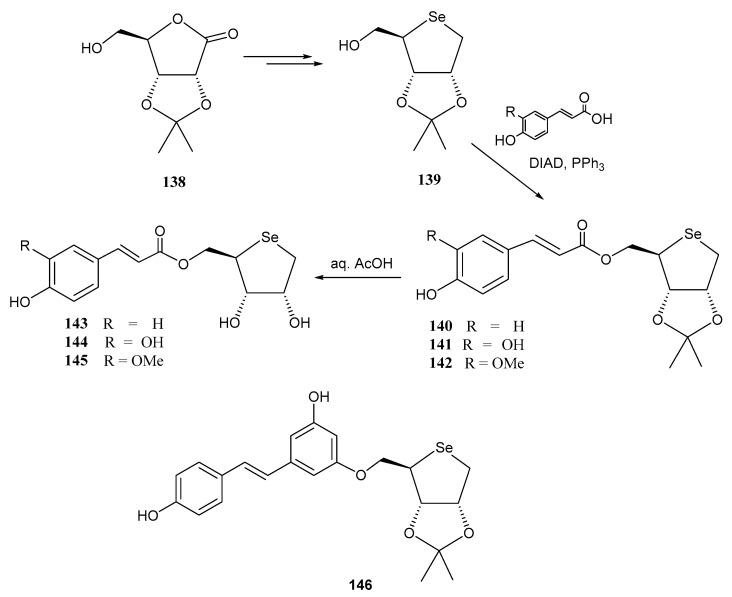
Preparation of selenosugar-phenolic acid conjugates through a Mitsunobu reaction.

Selenosugars have been conjugated with hydroxycinnamic acids, known for their potent antioxidant agents, via a Mitsunobu reaction to achieve synergic effects [103]. Initially, the authors intended [104] to use l-sugars (the C-4 epimer of **139**), obtained from d-ribose through a 6-step synthetic pathway: isopropylidene protection of the C-2 and C-3 positions, TBDPS protection at C-5, reduction of latent aldehyde, di-*O*-mesylation, nucleophilic displacement with NaHSe and acidic removal of TBDPS protecting group. However, unexpectedly, when the C-4 epimer of **139** was subjected to the Mitsunobu reaction with monoaceytlated hydroquinone, a configurational inversion occurred at C-4, resulting in a d-sugar instead [104]. Alternatively, d-configured selenosugar **139** can be obtained in a 5-step methodology starting from *O*-protected d-ribonolactone **138** [104]: mesylation of C-5 position, inversion of configuration on C-4 promoted by KOH, TBDPS-protection of C-5, reduction of the lactone moiety, di-*O*-mesylation, nucleophilic displacement with NaHSe, and C-5 deprotection. Subsequently, *p*-coumaric, caffeic and feluric acids were attached to the free OH at C-5 through a Mitsunobu reaction (giving compounds **140**–**142**) [103,104], in the presence of diisopropyl azodicarboxilate (DIAD) and PPh_3_ (Scheme 31).

Final deprotection of the isopropylidene protecting group under acidic conditions afforded conjugates **143**–**145**. These conjugates exhibited notable free-radical scavenging properties, reduced toxicity at concentrations up to 100 µM, and promising wound-healing properties in keratinocytes (in vitro scratch wound model), making them potential cosmeceutical ingredients [103,104]. Unprotected derivatives **144** and **145**, derived from *p*-caffeic and ferulic acids, demonstrated dose-dependent healing activity, at lower doses compared to the corresponding unconjugated cinnamic acids. The authors did not find a correlation between a higher cellular uptake and the wound healing properties, suggesting interaction with outer cell membrane components [103].

Using a similar approach with the C-4-epimer of **139**, conjugate **146** was synthesized [105] using diacetylated resveratrol in a Mitsunobu-type reaction (Scheme 31). *trans*-Resveratrol, a natural phytoalexina found in grapes and red wine, exhibits numerous biological properties like antioxidant, anti-inflammatory, cardio- and neuroprotective, and antidiabetic effects [106]. Caffeic acid and resveratrol conjugates **144** and **146** were loaded on a hydroxyl film to develop a pH-sensitive delivery system for accelerating skin wound healing. For this purpose, a copolymer comprised of poly(ethyleneglycol diacrylate) (PEGDA) and poly(hydroxyethyl methacrylate) (HEMA), in a 1:4.2 molar ratio, was used. Compound **144** was released at pH 7.4, suitable for acute wounds, whereas resveratrol-containing **146** was released at pH 9.6, optimal for chronic wounds [105]. The pH-depending selective release was attributed to weak interactions between the selenoconjugates and the hydroxyl groups of HEMA [105].

**Scheme 32:**
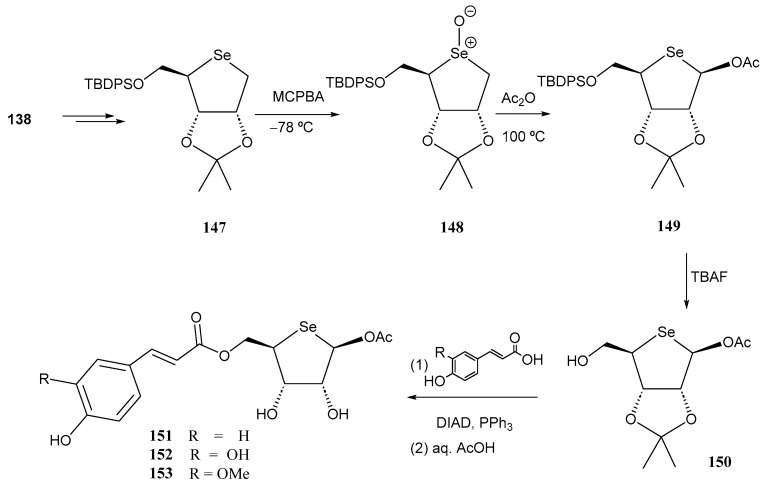
Preparation of the second-generation selenosugar-phenolic acid conjugates.

The same group recently developed [107] a second generation of selenosugar-cinnamic acid conjugates as cosmeceutical agents, introducing an acetoxy group at the pseudo-anomeric position to enhance cellular uptake. This structural feature was incorporated via a seleno-Pummerer rearrangement of selenoxide **148** upon heating in Ac_2_O (Scheme 32). This reaction has been used extensively by Iwaoka’s group for accessing selenonucleosides [108] (see Section 3.2). Subsequent silyl-*O*-deprotection, followed by Mitsunobu reaction with the same phenolic acids as described in Scheme 32, and final *O*-deprotection furnished conjugates **151**–**153**.

Compared to the first generation counterparts, the acetoxy derivatives **151**–**153** displayed a dose-dependent cytotoxicity, potentially affecting mitochondria redox activity at concentrations above 25 µM [107]. Among these, **153** exhibited a 7.5-fold increase in cell membrane permeability compared to **145**, which lacks the acetoxy group (HaCaT cells). The caffeoyl derivative **152** showed the strongest protective effect against H_2_O_2_, while selenosugars derived from *p*-coumaric and ferulic acids (**151** and **153**) demonstrated pro-oxidant properties.

Using a percutaneous absorption assay, first generation compounds, without the acetoxy group, generally exhibited higher skin penetration [107]. Compound **144** displayed the fastest penetration rate. However, **152** (derived from caffeic acid) was the only second-generation compound found in the receptor fluid layer.

### 3.2. Selenonucleosides

The incorporation of selenium into nucleosides has been proposed as a strategy to modulate or enhance their anticancer or antiviral properties [10]. Herein we focused specifically on replacing the endocyclic oxygen atom in the carbohydrate moiety with selenium. Previous studies exploring selenium substitution within the nitrogen base have been comprehensively reviewed [10].

**Scheme 33:**
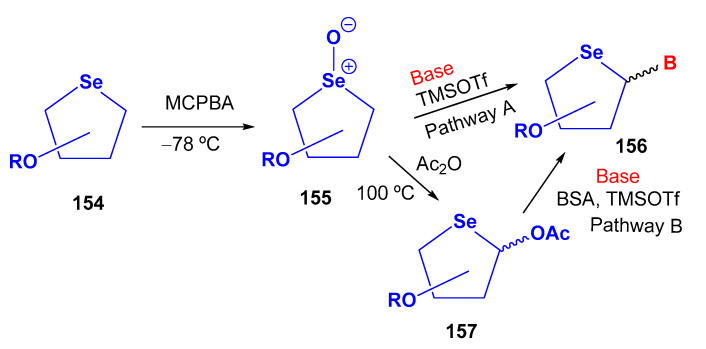
General procedure for the preparation of selenonucleosides.

Most reported examples of selenonucleosides describe the synthesis of the 4′-selenosugar moiety **154** via a double nucleophilic displacement reaction using in situ generated NaHSe on a di-*O*-mesylated derivative, as illustrated in Scheme 27. Subsequently, the nitrogen base is usually introduced into the selenosugar framework through two main strategies (Scheme 33). Pathway A: treatment of a selenoxide (**155**) directly with a silylated nitrogen base in the presence of TMSOTf and Et_3_N. Pathway B: via seleno-Pummerer rearrangement with Ac_2_O followed by *N*-glycosylation with a nitrogen base (Vorbrüggen glycosylation). In this approach, the selenoxide undergoes heating in the presence of Ac_2_O to produce the acetoxy derivative **157**, that acts as the glycosyl donor. The acetoxy intermediates then react with the appropriate nitrogen base in the presence of *N*,*O*-bis(trimethylsilyl)acetamide (BSA) and TMSOTf (Scheme 33).

**Scheme 34:**
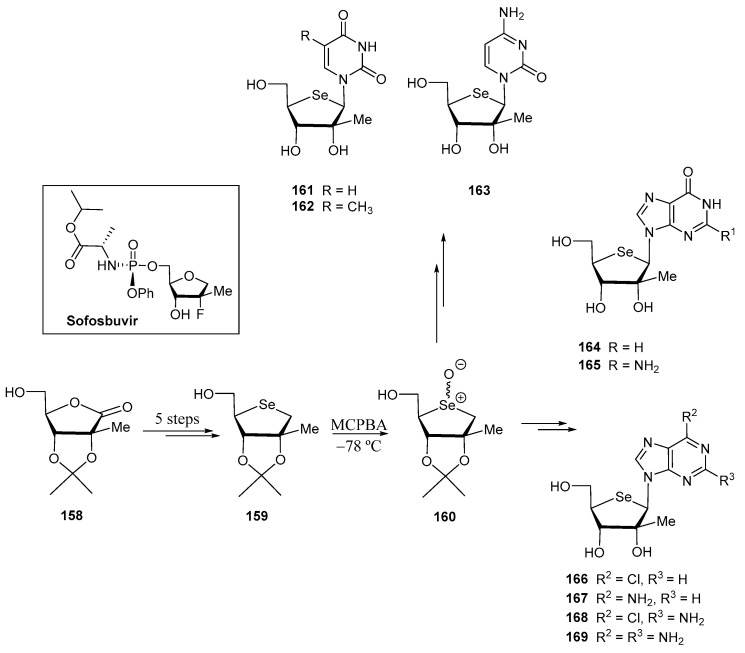
Preparation of 2′-C-methyl-4′-seleno pyrimidines and purines.

In the search for novel anti hepatitis C virus agents (HCV), Jeong and co-workers prepared Se-analogues of Sofosbuvir, an oral drug that is prescribed for the treatment of chronic HCV [109]. It was claimed that the presence of selenium might increase the lipophilicity compared to the 4′-oxo nucleosides, thus enabling these compounds to be transported across cell membranes; furthermore, the bulkier character of the selenium atom can also affect the nucleoside conformation [109]. Commercially available 2-C-methyl-d-ribono-γ-lactone **158** was transformed into the key 4′-selenofuranose **159** in a 5-step procedure, which included basic-promoted epimerization at C-4′ and nucleophilic displacement of a transient di-*O*-mesylate by selenide as the key steps. Then, two families of selenonucleosides were obtained [109] from **159** (Scheme 34): 4′-selenopyrimidine and purine nucleosides. The former family (**161**–**163**) was obtained following the pathway A of Scheme 33; MCPBA-promoted oxidation of **159** to give the glycosyl donor **160** followed by treatment with the appropriate pyrimidine base, TMSOTf and Et_3_N furnished the corresponding *O*-protected nucleosides in a roughly 1:2.5 α:β ratio [109]. Deprotection afforded derivatives **161**–**163** (Scheme 34).

For purine nucleosides, incorporation of the nitrogen base proceeded more efficiently using the Vorbrüggen glycosylation (pathway B of Scheme 33). Following this methodology, nucleosides **164**–**169** were obtained.

2D-NOESY experiments and X-ray crystallographic studies demonstrated that the selenofurane backbone of 2′-C-methyl selenonucleosides adopts a North conformation (C2′-exo/C-3′-endo), the same as the 4′-oxo derivatives, and opposed to the unusual conformation exhibited by 4′-selenoribofuranosyl nucleosides (South conformation, C2′-endo/C3′-exo) [110]. The authors claimed that probably, the sterical hindrance of the C2′-Me counteracted the effect of the selenium atom [109].

**Figure 12:**
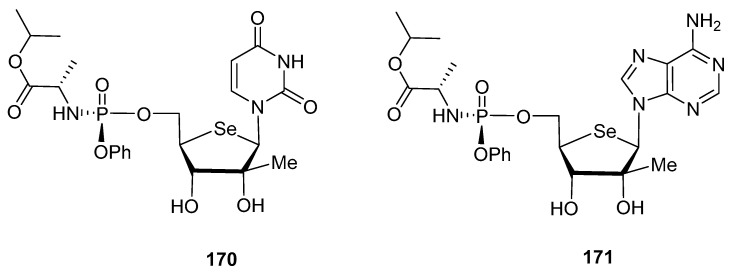
Structure of 5′-phosphoramidate-selenonucleosides **170** and **171**.

With the aim of mimicking the structure of Sofosbuvir, the authors also incorporated [109] a phosphoramidate residue at C-5′ position, affording compounds **170** and **171** (Figure 12), considered as prodrugs. The phosphoramidate moiety is incorporated to both increase the cell uptake, and to avoid the rate-determining 5′-monophosphorylation step by cellular kinases. Unfortunately, none of the 2′-C-methyl selenonucleosides showed relevant anti-HCV activity at the highest concentration tested (100 µM).

**Scheme 35:**
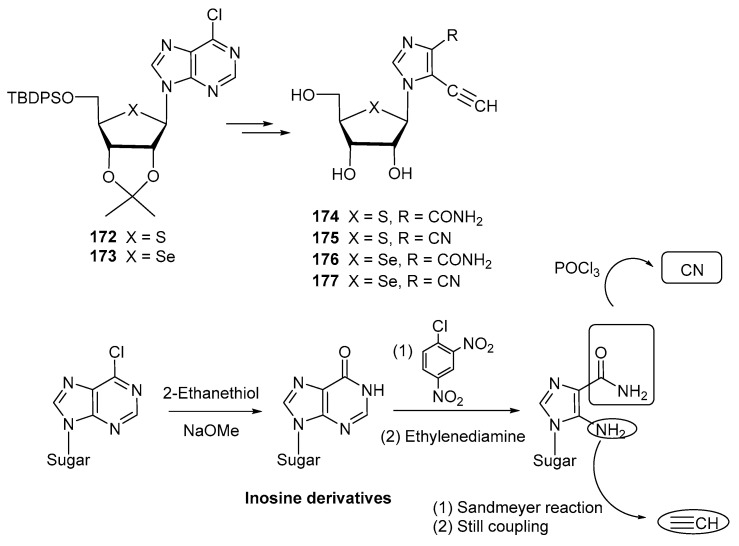
Preparation of imidazole thio- and selenonucleosides **174**–**177**.

Other nucleosides designed as antiviral agents are depicted in Scheme 35. Minakawa and co-workers envisioned [111] the synthesis of imidazole-derived thio- and selenonucleosides **174**–**177**, with potential anti-dengue virus (DENV) activity. The primary goal pursued by using an endocyclic chalcogen atom (S, Se) was to reduce the intrinsic cytotoxicity of their 4′-oxo analogues. Starting from 6-chloropurine nucleosides **172** and **173**, the purine moiety was degraded into the imidazole scaffold by first, conversion into the corresponding inosine derivatives with 2-ethanethiol in basic medium, followed by subsequent treatment with 1-chloro-2,4-dinitrobenzene and ethylenediamine [111].

The amido moiety was dehydrated with POCl_3_ to give a cyano motif (**175**, **177**), whereas the ethynyl functionality was obtained by subjecting the amino group on the imidazole to a Sandmeyer reaction, followed by a Stille-type coupling with the appropriate stannane derivative (Scheme 35). The compound with the highest capacity of inhibiting DENV replication was the thionucleoside **175**; although sensibly less potent than the analogous 4′-oxonucleosides (up-to-24 fold-less potent), the undesired cytotoxicity was considerably reduced (up to 40-fold) [111]. This compound exhibited virtually the same potency as ribavirin, a 1,2,4-triazolyl nucleoside used as a positive control.

**Figure 13:**
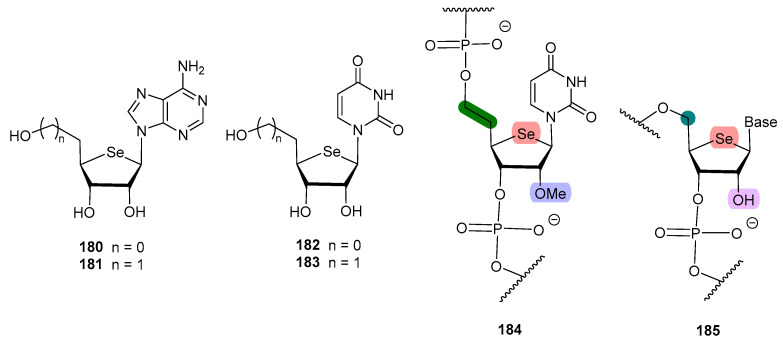
Selenonucleosides and their homologous isomers.

It has been hypothesized that the lack of antiviral activity exerted by some 4′-selenonucleosides could be attributed to either a change of the sugar reside conformation or to the bulky character of the selenium atom; this might hinder the kinase approach, thus preventing the phosphorylation at C-5′ position [112]. In order to prove this issue, Jeong and co-workers designed [112] 5′-homo-4′selenonucleosides, where the elongation of the exocyclic residue in one carbon might alleviate the sterical hindrance (**181**, **183**, Figure 13).

**Scheme 36:**
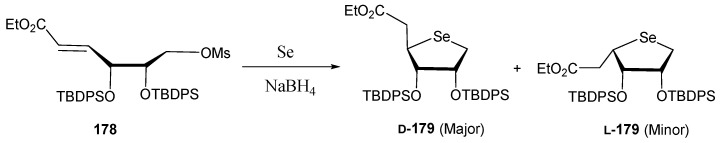
Se-Michael reaction for accessing 4′-selenofuranoses.

For this purpose, they used an innovative approach for accessing the selenosugar scaffold; instead of using the typical nucleophilic substitution on di-*O*-mesylated intermediates, a Se-Michael reaction was applied for the first time for constructing a carbohydrate backbone [112]. The use of bulky protecting groups allowed a good facial selectivity to achieve the d-sugar over the l-counterpart (Scheme 36).

The results align with the initial hypothesis, as derivatives **181** and **183** exhibited potent anti-herpes simplex virus (HSV-1) activity (EC_50_ = 2.9 and 2.3 µM, respectively) [112], whereas 4′-selenonucleosides **180** and **182**, previously synthesized by the same authors [110,113], turned out to be inactive (EC_50_ > 100 µM).

In order to develop oligonucleotides with therapeutic interest, their stability towards ribonucleases must be improved, by hindering the hydrolysis of the phosphodiester bonds. The most widely used procedure is to incorporate a methoxy group at the C-2′ position of the sugar residue. Jeong and co-workers combined the homologation approach [112] to maintain the biological activity of the selenonucleoside with a methoxy group at C-2′, and prepared oligonucleotides containing the 5′-homo-4′-selenouridine fragment (**184**, Figure 13) [114]. This modified selenonucleoside was proved to reduce the capacity of the oligonucleotide to form duplexes, probably because of the free rotation enabled by the extra carbon, and had reduced thermal stability. However, a remarkable increase in its stability towards nuclease-mediated digestion was found; tentatively, the homologated structure caused a reduced affinity with the enzyme [114].

Interestingly, Minakawa and co-workers prepared 4′-selenoRNA **185** (uridine, cytidine, adenosine, guanosine), with no homologation, and without a masked hydroxyl group at C-2′ [115]. The key synthetic intermediates were the corresponding phosphoramidites, which afforded the 4′-selenoRNA using a DNA/RNA synthesizer. These compounds enhanced the formation or RNA duplexes, and exhibited a high stability towards endonucleases, despite the presence of the free 2′-OH moiety [115].

**Scheme 37:**
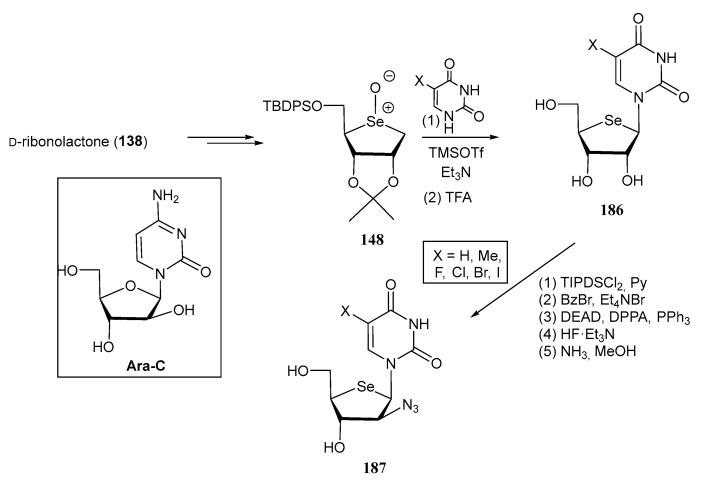
Synthetic pathway for the preparation of selenonucleosides **187**.

Many selenonucleosides also exhibit notable anticancer properties. In this context, d-arabino-configured Arabine-C (cytarabine), is a well-known nucleoside widely used as a chemotherapeutic agent for the treatment of leukaemia and lymphomas. Isosteric derivatives bearing F and N_3_ motifs at the C-2′ position have demonstrated noteworthy antiviral and anticancer properties [116]. These structural templates inspired Jeong and co-workers [116] to develop an extensive library of 2′-substituted-4′-selenoarabinofuranosyl pyrimidines. Thus, starting from d-ribonolactone **138**, and following the pathway A illustrated in Scheme 33, the pyrimidine moiety was installed on the glycosyl donor **148** to give selenonucleosides **186**. Selenonucleosides **187**, with d-*arabino* configuration and bearing an azido motif on C-2′ were accessed from **186** in 5 steps: TIPDS protection at C-3 and C-5 positions of the sugar residue, benzoylation of the nitrogen base, Mitsunobu reaction with dipheynylphosphoryl azide (DPPA), and subsequent removal of silyl and benzoate protecting groups (Scheme 37).

**Scheme 38:**
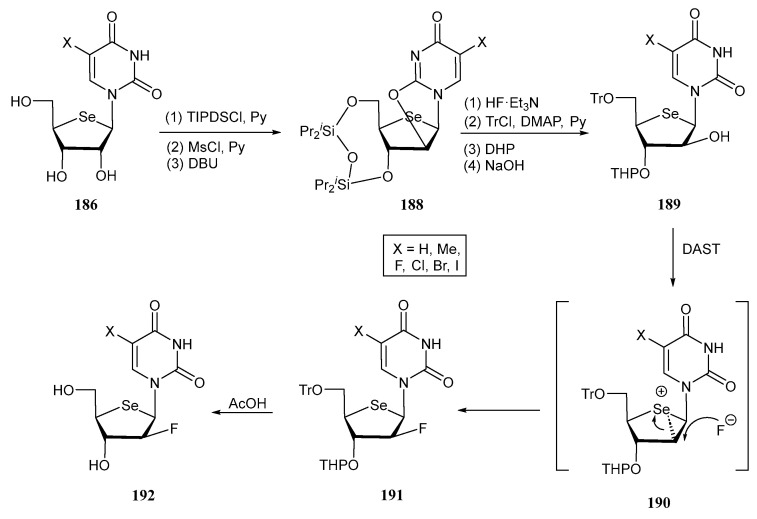
Synthetic pathway for the preparation of selenonucleosides **192**.

Fluorinated selenonucleosides **192** were prepared from the fully unprotected derivative **186** as outlined in Scheme 38 [116]. After TIPDS protection, mesylation at the C-2′ position and DBU-treatment afforded anhydro nucleoside **188**, with configurational inversion at C-2′. Subsequent *O*-deprotection, followed by chemoselective tritylation of the primary alcohol, regioselective protection at C-3′ and hydrolysis of the anhydro moiety gave **189**. Finally, fluorination on C-2′ with retention of the configuration was accomplished with diethylaminosulfur trifluoride (DAST) through a double S_N_2 reaction involving the selenium atom, followed by acidic deprotection to afford **192**.

**Scheme 39:**
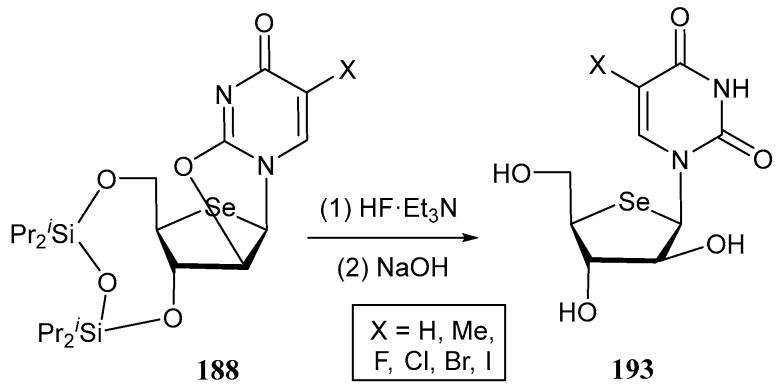
Synthetic pathway for the preparation of selenonucleosides **193**.

The third family of 2′-substituted-4′-selenoarabinofuranosyl pyrimidines prepared by Jeong and co-workers consisted of derivatives **193**, as shown in Scheme 39 [116]. Deprotection of C-3′ and C-5, followed by hydrolysis of the anhydro moiety in **188** afforded d-arabino-configured selenonucleosides **193** with an OH at the C-2′ position.

**Scheme 40:**
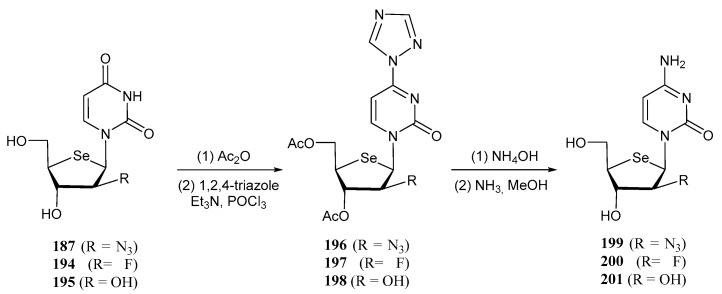
Synthetic pathway for the preparation of selenonucleosides of cytidine-type.

Selenonucleosides of the cytidine type were obtained using the approach illustrated in Scheme 40. Derivatives **187**, **194** and **195** (uracyl selenonucleosides, X = H) were acetylated at C-3′ and C-5′ positions, and treated with 1,2,4-triazole to furnish unstable triazole derivatives **196**–**198**. Final treatment with NH_4_OH in dioxane and methanolic NH_3_ gave cytidines **199**–**201**.

Compounds **187**, **194**, **195** and **199**–**201** were tested in vitro as potential antiproliferative agents against a panel of 6 tumour cell lines. Although most of them were either inactive at the highest concentration tested (100 µM), or moderate, compound **200** turned out to be a potent antiproliferative agent, with IC_50_ ranging from 0.14–1.1 µM, with a better profile than Ara-C, used as a positive control [116].

**Figure 14:**
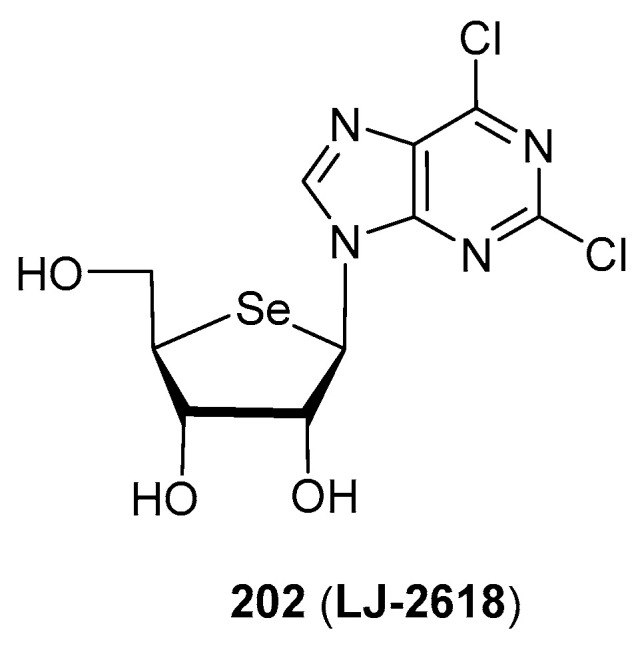
Structure of the anticancer selenonucleoside **LJ-2618**.

A very remarkable example of anticancer activities exhibited by selenonucleosides is 4′-selenofuranosyl-2,6-dichloropurine (**202**, **LJ-2618**, Figure 14). This compound was tested [117] in vitro and in vivo (xenograft mouse model) against drug-sensitive and paclitaxel-resistant prostate cancer cells (PC-3 and PC-3-Pa, respectively). Although less potent than paclitaxel, compound **LJ-2618** maintained the same activity in both drug-sensitive and drug-resistant cells (IC_50_ = 11.6 and 11.7 µM, respectively); paclitaxel exhibited a 108-fold impairment of activity in PC-3-Pa. Moreover, mice implanted with PC-3-Pa cells showed a significant diminishment of the tumour volume after 29 days of treatment with **LJ-2618** (3 or 10 mg/Kg, 3 times/week) of 50.0 and 66.1%. These results strongly contrast with those exhibited by paclitaxel (26.6% reduction of the tumour, 5 mg/Kg treatment) [117]. This compound was shown to induce a G_2_/M cell cycle arrest via down-regulation of the Skp2 (S-Phase Kinase Associated Protein 2) expression in PC-3-Pa cells, a hitherto unknown target for selenonucleosides [117].

**Figure 15:**
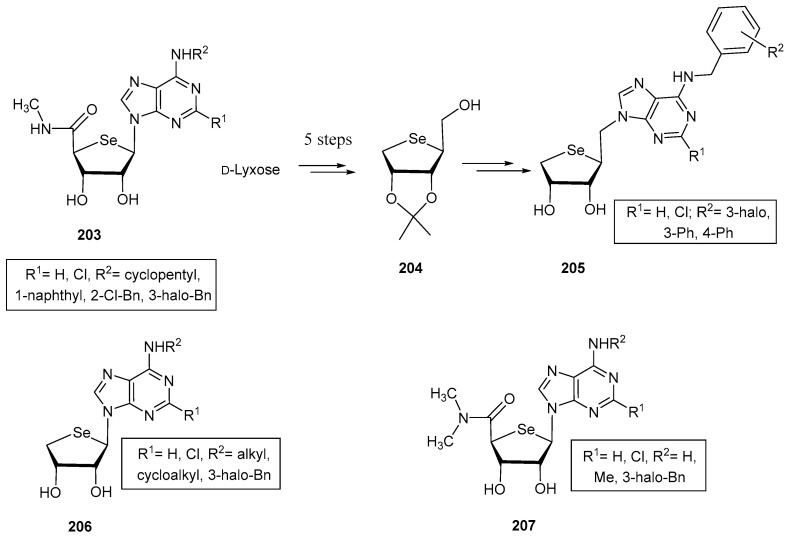
Structure of 5′-*N*-methylcarbamoyl-4′-selenoadenosines and truncated/homologated analogues.

A template that has been extensively studied is 4′-selenoadenosine in connection with peroxisome proliferator-activated and A_3_ adenosine receptors (PPAR and A_3_AR, respectively). PPARs (classified as PPARα, γ and δ, depending on the tissue distribution and specific function), have a key role in maintaining the metabolic homeostasis, with a direct involvement in the metabolism of lipids and glucose, adipogenesis, and also in inflammatory responses [118]. Accordingly, PPARs are interesting therapeutic targets for metabolic disorders and inflammatory diseases, like inflammatory bowel disease. Jeon, Noh and co-workers developed a series of selenoadenosine nucleosides (**203**, Figure 15) by modification of the substituents at C-2 (H, Cl) and C-6 positions N^6^-(cycloalkyl, aryl, halobenzyl) of the base [119]. Interestingly, derivative **203**, decorated with a Cl atom at C-2 and a 3-iodobenzyl moiety at N^6^ exhibited an enhanced antagonist activity against PPARδ compared to its 4′-oxo and 4′-thio counterparts. This behaviour was attributed to the unusual South conformation; docking simulations confirmed additional hydrogen bonding within the hydrophobic pocket of the enzyme, what was lacking in its other chalcogen isosters [119].

In subsequent investigations, structures **205** were obtained (Figure 15). These compounds are truncated homologated analogues of **203**, where the carboxamido motif has been eliminated, and an extra carbon has been introduced at C-1′ position [120]. These selenoucleosides were obtained using the selenofuranose **204** as the key synthetic intermediate (Figure 15), which in turn was obtained starting from d-lyxose in 6 synthetic steps: acetal protection of C-2 and C-3 positions, chemoselective protection of the primary alcohol, reduction of the latent aldehyde, di-*O*-mesylation, nucleophilic displacement with in situ generated selenide and primary *O*-deprotection. The nucleobase was introduced in this case via an S_N_2 reaction prior mesylation of **204 [120]**. Again, the best compound in the series within **205** incorporated a Cl atom at C-2 and a 3-iodobenzyl moiety at N^6^. This compound behaved as a potent PPARγ partial agonist (Ki = 2.8 μM) and a PPARδ antagonist (Ki = 43 nM). Additionally, it improved the production of adiponectin, a hormone secreted for controlling the metabolism of fatty acids and glucose, increasing sensitivity to insulin. This effect demonstrates that the synergic modulation of both receptors can lead to promising agents for the treatment of metabolic disorders, specifically those associated with hypoadiponectinemia [120].

Bioisosteric replacement of oxygen and sulfur in previous nucleosides by selenium led to A_3_AR agonists, a feature that can allow the development of new drugs for the treatment of inflammatory and autoimmune diseases. In this context, derivative **205** bearing a H at H-2 and a N^6^-3-iodobenzyl moiety (Figure 15), behaved as a subnanomolar agonist of such receptor (K_i_ = 0.57 nM) [121]. This represents an outstanding selectivity compared to A_1_AR and A_3_AR (selectivity index > 800 and 1900, respectively). Structural analysis revealed that these selenonucleosides exhibit again a South furanose puckering, and a syn orientation of the nucleobase; surprisingly, previous A_3_AR agonist exhibit the complete opposite conformational preference. The lead compound also showed an improved inhibition of the MCP-1 induced microglial chemotaxis, suggesting potential anti-stroke properties [121].

Further Structure-Activity studies conducted on adenosine receptors led to the preparation of selenonucleosides **206**, with a truncated structure [122], and **207 [123]**, with a disubstituted nitrogen atom on the carboxamido motif (Figure 15). Compounds **206** exhibited strong binding to A3AR, within the nanomolar range. Surprisingly, the two most potent compounds (R^1^ = Cl) incorporated alkyl groups on R^2^ positions (Me and cycloalkyl, K_i_ = 5.2 and 5.7 nM). Computational calculations demonstrated that the South puckering of the carbohydrate residue allowed a closer location within the binding site of OH-3 to Thr94, compared to 4′-oxo nucleosides [122]. Furthermore, an agonist effect was observed, unlike their truncated oxygen and sulfur counterparts, which behaved as antagonist of this receptor.

Remarkably, the addition of a second methyl to the carboxamido moiety (**207**) shifted the activity of the 4-‘selenonucleosides to antagonism, with medium to high binding affinity [123]. The best compounds lacked a chlorine atom at the 2-position of the nucleobase, and the lead one was decorated with a 3-iodobenzyl scaffold at N^6^-position (K_i_ = 22.7 nM). The same behaviour, and similar binding affinities were found for isosteric 4′-oxo and 4′-thionucleosides. Theoretical calculations revealed the essential role of the NH moiety for exhibiting agonist activity [123].

### 3.3. Selenoglycosides

**Figure 16:**
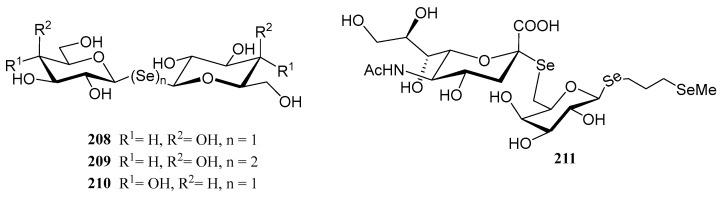
Representative selenoglycosides for structural studies.

The substitution of the oxygen atom in the glycosidic bond with selenium has emerged as a prominent strategy in glycomimetic synthesis. This isosteric modification enables the development of biomimetics with enhanced metabolic stability compared to their natural counterparts, while maintaining dynamic and conformational properties similar to those of thioglycosides [124]. Selenoglycosides, through ^77^Se-NMR, X-ray crystallography or calorimetry titrations, are widely used in structural studies [124,125,126,127], particularly for gaining deeper insights into carbohydrate recognition by protein receptors, such as lectins. Notable examples of these glycomimetics include the seleno- and diselenodiglycosides **208**–**210** [124,126,127], which have been studied for their binding to human galectins 1- and 3, as well as Se-sialoside **211**, a mimetic of sialyl α(2,6) and α(2,3)-galactose epitopes, relevant for investigating glycan-pathogen interactions (Figure 16). Numerous selenoglycosides also exhibit relevant biological properties; for instance, Comini and co-workers evaluated [128] an ample panel of selenoglycosides as potential antiparasitic agents against trypanosomiasis; the lead compounds exhibited activity within the submicromolar range, and good selectivity.

**Figure 17:**
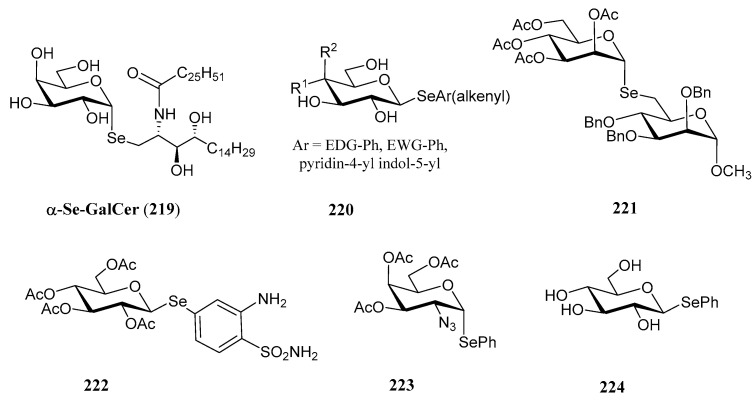
Relevant selenoglycosides.

**Scheme 41:**
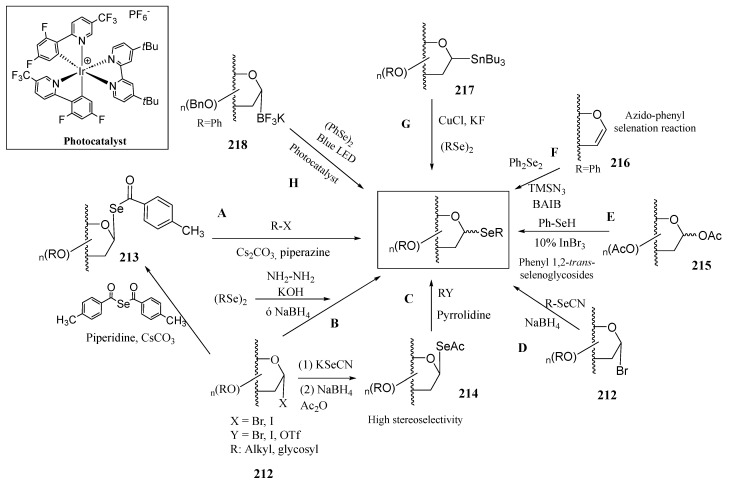
Main synthetic procedures for the preparation of selenoglycosides.

The growing interest in selenoglycosides has driven the need for practical and stereoselective synthetic methods. Most of these approaches are based on nucleophilic displacements carried out by Se-based nucleophiles (Scheme 41) on glycosyl halides (**212**), triflates, acetates (**215**) and glycals (**216**). For instance, the treatment of glycosyl halides with *p*-methylselenobenzoic anhydride in the presence of Cs_2_CO_3_ and piperidine, generates *p*-methylselenobenzoate in situ, which then undergoes a S_N_2 reaction at the anomeric position, with inversion of configuration, yielding *p*-methylbenzoylselenoglycoside **213** (Scheme 41, pathway A). Further reaction with alkyl halides, including those containing sugar residues, under basic conditions enables the preparation of the corresponding selenoglycosides [125,129]. Using this methodology as one of the key steps, Murphy and coworkers accomplished [129] the preparation of **219** (Figure 17), the Se-isoster of the immunostimulant α-GalCer. In their synthetic pathway, TiCl_4_ was used to isomerize an equatorial β-selenoglycoside into its axial α-counterpart.

**Scheme 42:**
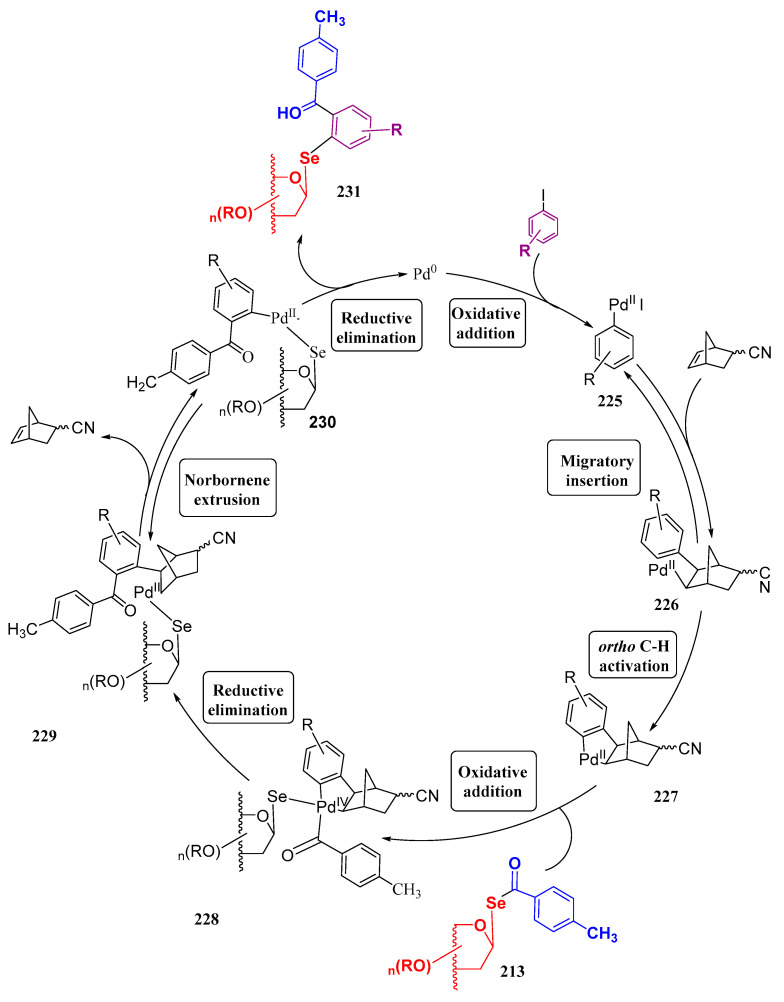
Catalytic cycle of the Castellani-type reaction for the synthesis of (hetero)aryl selenoglycosides.

A transient *p*-methylselenobenzoate intermediate was also used [130] in cross-coupling reactions by treatment with heteroaryl and alkenyl halides using a Pd-based catalyst (PdG3 XantPhos) under mild conditions. This process furnished the unprecedented formation of a C(sp^2^)-Se bond, like in **220** (Figure 17). This methodology proved to be compatible with a variety of functional groups, including aldehydes, ketones, or nitriles, among others. However, it was claimed [131] that, despite being an elegant approach, it lacks atom economy, as the acyl moiety in **213** is not incorporated into the final structure. This issue was overcome by Liang and coworkers by using modified conditions of the Castellani reaction (Scheme 42), originally envisioned for the synthesis of arenes, and based on Pd/norbornene (NBE) cooperative catalysis [132]. They reported the unprecedented use of two-component Castallani-type reaction for coupling *p*-methylbenzoylselenoglycosides **213** with (hetero)aryl iodides to furnish selenoglycosides upon formation of a new C(sp^2^)-Se bond, and concomitant Se–C(=O) breaking. A similar procedure was also employed for accessing the corresponding thioisosters. This strategy proved to have a broad scope, being effective in *O*-protected pyranoses and furanoses (ribose, glucose, mannose, galactose, xylose, arabinose), including disaccharides like cellobiose and maltose, and tolerates a variety of functional groups (Ac, Piv, Bn, TBDPS, Me). A tentative catalytic cycle (adapted from the one reported for thioglycosides) is illustrated in Scheme 42. (Pd(MeCN)_2_Cl_2_ is used as the Pd(II) source, and P(*p*-Cl-C_6_H_4_)_3_ as the ligand; the formation of the selenoglycoside is postulated to take place through seven steps [131]: oxidative addition of Pd(0) into aryl iodides, migratory insertion of 5-norbornene-2-carbonitrile, ortho C-H activation to give the five-membered palladacycle **227**, oxidative addition of *p*-methylbenzoylselenoglycoside **213**, reductive elimination, norbornene extrusion, and a second reductive elimination to give selenoglycoside **231**, where no loss of atoms has taken place. The main concerns of this approach are the high temperatures (95–100 °C) and the long reaction times (16 h).

**Scheme 43:**
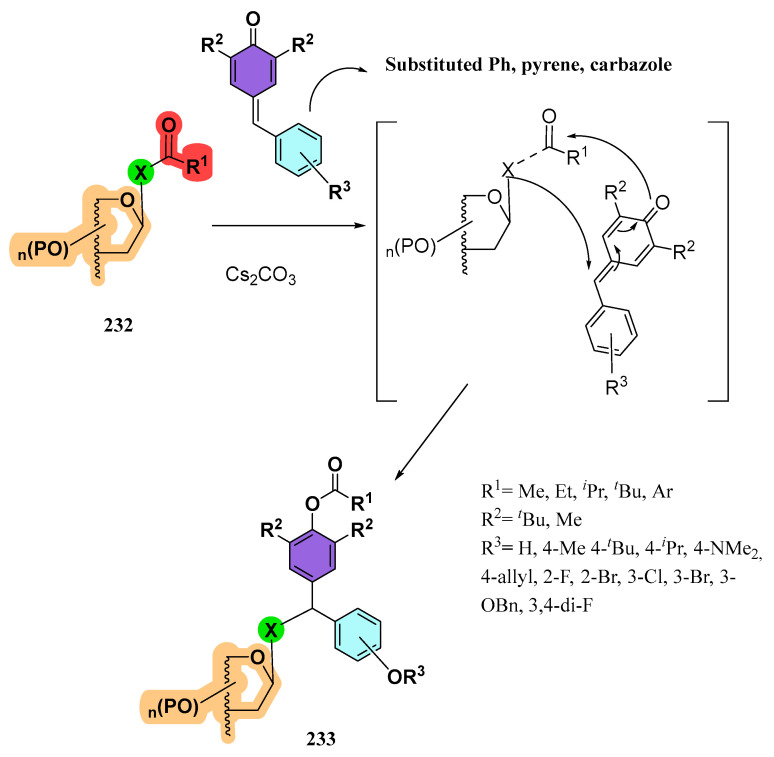
Preparation of unsymmetrical *gem*-diarylmethylthio(seleno)glycosides.

Azeem and Mandal described [133] the atom-economic and gram-scale synthesis of unsymmetrical gem-diarylmethyl(thio)seleno glycosides. For this purpose, glycosyl thio(seleno) acetates and other acylated derivatives **232** (analogues to **213**) were treated with Cs_2_CO_3_ and *p*-quinone methides (*p*-QMs). It was hypothesized that Cs_2_CO_3_ promotes the cleavage of the X-C(=O) bond, generating a transient thiolate/selenolate that undergoes a 1,6-addition on the *p*-QMs and an acyl transfer via a concerted mechanism (Scheme 43). Reactions proceeded smoothly (rt), with short reaction times (30 min-2 h), and the selenoglycosides **233** were isolated in a roughly 1:1 diasteromeric ratio.

1,2-trans-Selenoglycosides (alkyl, aryl, glycosyl) can be obtained [134] through the nucleophilic attack of selenides (Scheme 41, Pathway B), which are generated via the reductive cleavage of symmetrical diselenides. These selenides then react with per-*O*-acetylated glycosyl bromides under phase transfer catalysis. Using a similar approach, Oscarson and coworkers reported [135] the preparation of selenoglycosides bearing a fluorine atom as useful tools for studying protein-carbohydrate interactions with ^19^F and ^77^Se-NMR spectroscopy.

Another approach to prepare selenoglycosides from glycosyl halides 212 is illustrated in Scheme 41, Pathway C. Treatment with KSeCN affords glycosyl selenocyanates, typically with inversion of configuration. Reduction of the selenocyanato motif with NaBH_4_ generates a configurationally stable selenide, which is subsequently trapped through acetylation (214). Further reaction with alkyl halides under basic conditions leads to the formation of the corresponding selenoglycoside [136] in good yields, and short reaction times (10–20 min). Disaccharides, like 221, were also obtained with this procedure.

Glycosyl bromides 212 were also treated with aryl selenides, generated via the NaBH_4_-mediated reduction of the corresponding aryl selenocyanates (Scheme 41, Pathway D). This strategy was used by Angeli and coworkers [137] to develop novel sulfonamides bearing a selenoglycoside linkage, like 222, designed for targeting seizures associated with glucose transporter type 1 deficiency syndrome (GLUT1-DS).

Reactions involving Se-based nucleophiles can also take place in per-*O*-acetylated aldoses, using benzene selenol and InBr_3_ as a moisture-stable Lewis acid (Scheme 41, Pathway E) [138]. This strategy affords 1,2-trans selenoglycosides, due to neighbouring group participation, in moderate to almost quantitative yields. The methodology is also applicable to the preparation of phenyl thioglycosides.

Nifantiev and coworkers reported [139] the heterogeneous and reproducible 2-azido-phenylselenylation of 3,5,6-tri-*O*-acetyl-d-galactal with Ph_2_Se_2_ and TMSN_3_ (Scheme 41, Pathway F) in the presence of the hypervalent iodine compound BAIB (PhI(OAc)_2_, [bis(acetoxy)iodo]benzene). This reaction afforded crystalline phenyl 3,4,6-tri-*O*-acetyl-2-azido-2-deoxy-1-seleno-α-d-galactopyranoside (223), used for the preparation of galactosamine building blocks. Conversely, the use of d-glucal did not proceed with stereoselectivity, resulting in a mixture of d-gluco and d-manno selenoglycosides. The use of flow chemistry allowed a reduction of side-products, and the reaction time, compared to the batch process [140]; optimised conditions led to the production of the above 2-azidoselenoglycoside in 1.2 mmol/h rate, affording the processing of 5 mmol of galactal over a 3 h-period.

Alternatively, Walczak and coworkers reported the stereoretentive synthesis of selenoglycosides starting from glycosyl tributyl stannanes and symmetrical diselenides in the presence of CuCl and KF (Scheme 41, Pathway G) [141]. No directing groups are required, and the reaction proceeds successfully with free hydroxyl groups (e.g., **224**), affording a broad scope that enabled the preparation of a vast library of compounds. It was hypothesized that the tributyl stannane undergoes a stereoretentive transmetallation reaction with CuCl, enabled by the presence of F^−^. Then, the glycosyl organocopper intermediate reacts with the diselenide, collapsing to the corresponding selenoglycoside [141]. The main limitations are the temperatures (110 °C) and hazardous organotin reagents.

The same group recently developed a procedure for the stereoretentive cross-coupling reaction of 2-deoxysugars bearing anomeric trifluoroborates. The main targets were C-arylation and etherification reactions under photoredox conditions (via a single electron transfer mechanism) [142]. As the only example of a selenoglycoside, they described the preparation of phenyl 2-deoxy-3,4,6-tri-*O*-benzyl-α-d-glucopyranoside with total retention of configuration (Scheme 41, Pathway H).

**Scheme 44:**
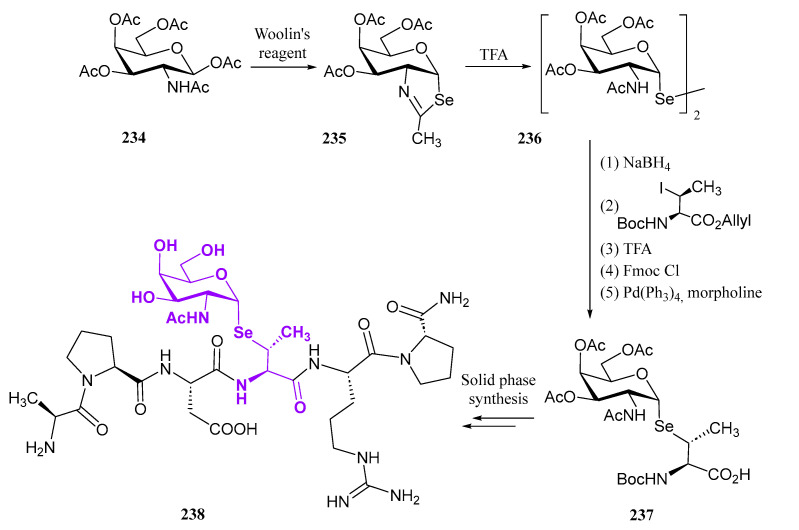
Synthesis of Se-GalNAc-glycopeptide as a tumour-associated antigen.

Corzana and coworkers reported [143] the preparation of S- and Se-mimetics of a GalNAc glycopeptide (compound **238**, Scheme 44) derived from mucin MUC1, an *O*-glycoprotein overexpressed in a series of tumours. This glycopeptide acts as a tumour-associated antigen, and thus, is potentially useful in the development of cancer vaccines. The substitution of oxygen by bulkier S and Se atoms increases the distance between the saccharide residue and the oligopeptide, and also alters the orientation of the glycosidic bond. These structural modifications allowed the glycopeptide **238** and its sulfur isoster to adopt a pre-organized conformation that improved binding to the MUC1 antibody [143]. The preparation of selenoglycoside **237**, a key synthetic intermediate latter subjected to solid-phase peptide synthesis, involved a nucleophilic displacement exerted by a selenium nucleophile (Scheme 44). Unlike previous examples, in this case the diselenide motif was located at the anomeric position. This was achieved starting from per-*O*-acetylated GalNAc, which upon treatment with Woolin’s reagent afforded a transient selenoamide that evolved to bicyclic 2-methylselenazoline **235** through a spontaneous intramolecular nucleophilic cyclization. Subsequent treatment with TFA furnished diselenide **236** which was then reduced and coupled to an iodinated derivative of l-threonine [143].

**Scheme 45:**
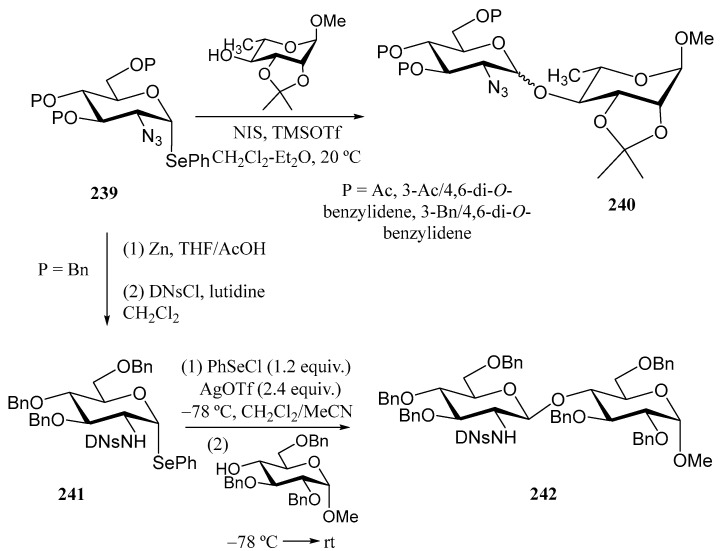
Use of selenoglycosides in glycosylation reactions.

Selenoglycosides are not only relevant for structural studies or for the development of bioactive compounds, but they are also valuable intermediates in organic synthesis, as they can be used as glycosyl donors with different alcohols (including partially protected saccharides) and promoters. This strategy has also been used in the preparation of complex oligosaccharides [144]. Phenyl α-selenoglycosides, obtained via an azido-phenyl selenation reaction from glycals as depicted in Scheme 41 (Pathway F) were activated using classical glycosylation conditions with NIS and TMSOTf, and treated with either primary or secondary (sugar-derived) alcohols (Scheme 45); in the latter case, disaccharides were obtained [145]. 2-Azido motifs are the precursors of amino and acetamido scaffolds, present in numerous bioactive glycans. When d-*gluco*-configured selenoglycosides were used (e.g., **240**), a good α:β selectivity was observed (3–5:1); interestingly, mannopyranosides afforded α-linked glycosides exclusively.

Alternatively, Li and co-workers converted the 2-azido functionality in **239** (*O*-benzyl protected) into 2-deoxy-2-(2,4-dinitrobenzenesulfonyl)amino (DNsNH) (compound **241**, Scheme 45) [146], which in turn can be treated with thioacetic acid and DMAP to give an acetamido motif. Activation of 2-deoxy-2-DNsNH phenyl selenoglycoside **241** with a combination of PhSeCl and AgOTf, followed by the addition of the glycosyl acceptor at low temperatures afforded disaccharide **242** with good β-stereoselectivity. This protocol proved to be efficient even with sterically hindered glycosyl acceptors, with reduced nucleophilic properties. The combination of **241** with sequential thioglycoside-based donors enabled the preparation of β-(1→6)-linked tri- and hexasaccharides with potential interest as antigens in the search for vaccines against microbial infections [146].

### 3.4. Miscellaneous Selenosugars

**Figure 18:**
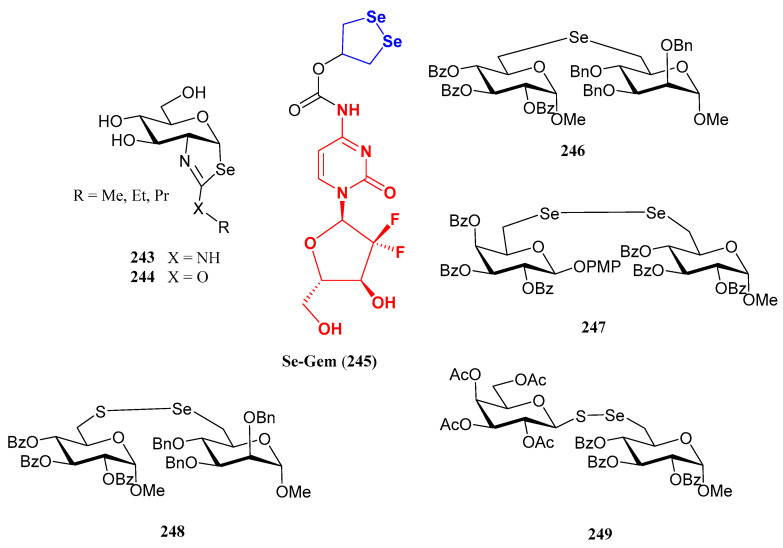
Miscellaneous selenosugars.

Other sugar mimetics containing selenium have been reported, besides selenosugars, selenonucleosides and selenoglycosides. Figure 18 shows some recent examples of such structures. Thus, López and co-workers reported [74] the preparation of sugar-derived bicyclic 1,3-selenazolines bearing either an alkylamino (**243**) or alkoxy (**244**) residue at the C-2 position of the heterocyclic motif. Such structures were designed as dual inhibitors of cholinesterases and O-GlcNAcase (OGA), two enzymes that are considered as validated targets against Alzheimer’s disease; it was postulated that the presence of a Se atom might contribute to maintain the redox homeostasis. Derivatives **243**, and particular the derivative with R = Pr exhibited the best bioactivities. This compound acted as a submicromolar selective inhibitor of butyrylcholinesterase (predominant in advanced stages of the disease; IC_50_ = 0.46 µM) and a nanomolar selective inhibitor of human OGA (IC_50_ = 53 nM), while showing no significant activity against glycosidases or cytotoxicity [74].

Fan’s group designed prodrugs derived from the antitumour drug gemcitabine and a 1,2-diselenolane moiety connected through a carbamate linker (**Se-Gem**, **245**, Figure 18) [147]. This compound, which acted as a suicide prodrug, improved the antiproliferative activity of gemcitabine (up to 6-fold, IC_50_ = 0.11–0.88 µM). It was activated by glutathione (GSH), releasing gemcitabine and the diselenide, depleting GSH and increasing the oxidative stress, what in turn induced cell-death by apoptosis [147].

Using *O*-protected monosaccharides decorated with a selenocyanato motif either at C-4 or C-6 position, Misra’s group accomplished the preparation of non-symmetrical and non-glycosidic pseudodisaccharides connected through a selenide (**246**) [148] or a diselenide (**247**) [149] tether (Figure 18). In both cases, the key step is the reduction of the selenocyanato group with hydrazine hydrate to give a transient and reactive sugar selenide, which traps a sugar iodide or triflate to **246**, or a symmetrical sugar diselenide to give **247**. Additionally, treatment of a 6-iodo sugar derivative with a symmetrical sugar diselenide, CS_2_ and Na_2_S·9H_2_O afforded a pseudo disaccharide with a S-Se linkage (e.g., **248**) [150]. When the leaving group is located on the anomeric position (acetobromo aldoses), reaction with sugar diselenides afforded compounds like **249**, with exclusive formation of the β-anomer. This methodology proved to be scalable, odourless and high-yielding [150].

## 4. Te-Containing Carbohydrates

Tellurium was first identified by von Reichstein in 1782, while the synthesis of the first organotellurium compounds (dialkyl tellurides) was achieved in 1840 by Wöher [151]. However, significant advances in the field of organotellurium chemistry did not occur until the 1980s, roughly a decade after organoselenium chemistry did [152]. This delay can be attributed to the fact that low-molecular weight organotellurium derivatives are typically volatile, malodorous and perceived as toxic substances [152]. Initially considered as an extension of Se-containing isomers, organotellurium chemistry has since unlocked new synthetic possibilities [153]. The synthesis and applications of organotellurium derivatives are currently being explored in a wide range of areas, including organo- and metal-based catalysis [154,155], biocatalysts [156], Material Science [157,158,159], and Medicinal Chemistry [160,161], among others.

### 4.1. Tellurosugars

When it comes to carbohydrate mimetics, the number of tellurium-containing saccharides is significantly smaller compared to their thio- and seleno counterparts. In these derivatives, tellurium has been incorporated in different positions of the carbohydrate skeleton, such as the endocyclic position, the aglycon residue, or other specific sites, like the C-5 position of xylofuranoses (protecting agents against oxidative stress in in vivo experiments) [162], or C-2 and C-6 positions of cyclodextrins (as GPx mimics) [163].

**Scheme 46:**
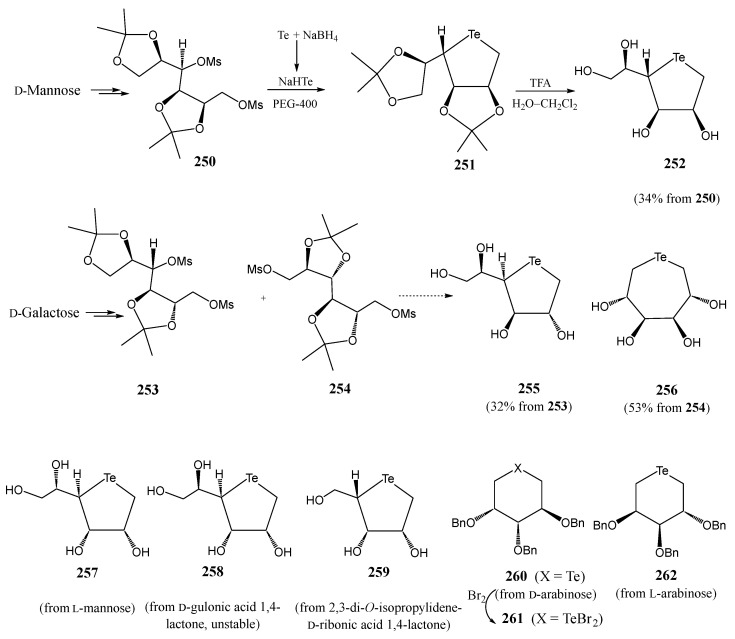
Preparation of tellurosugars by nucleophilic substitution on di-*O*-mesylalditols.

Inspired by the promising antioxidant properties of selenosugars, Schiesser and co-workers reported [11,164] the preparation of isosteric 4-tellurofuranoses (**252**, **255**, **257**–**259**), 5-telluropyranoses **260**–**262** and 6-tellurepane **256** as water soluble carbohydrate mimics. These compounds were accessed using naturally occurring carbohydrates with different configuration as starting materials. They key step of their syntheses involved a double nucleophilic substitution reaction on dimesylated alditols (e.g., **250**, **253**, **254**) exerted by freshly prepared NaHTe (Scheme 46). NaHTe was in turn obtained by reducing elemental Te with NaBH_4_. The nucleophilic displacement reaction was found to proceed more efficiently when polytethylene glycol (PEG-400) was used as solvent. Additionally, the choice of the protecting group played a crucial role in the outcome of the reaction. Whereas *O*-benzylated tellurosugars **260**–**262** could not be deprotected [164] to yield the corresponding unprotected carbohydrate mimics, acetals were successfully removed upon treatment with TFA. 5-Telluropyranose **260** was found to be highly unstable, even when preserved in the freezer, and spontaneously underwent decomposition, with release of elemental Te; in order to demonstrate the formation of **260**, it was transformed into dibromotellurium derivative **261**, upon reaction with Br_2_.

**Scheme 47:**
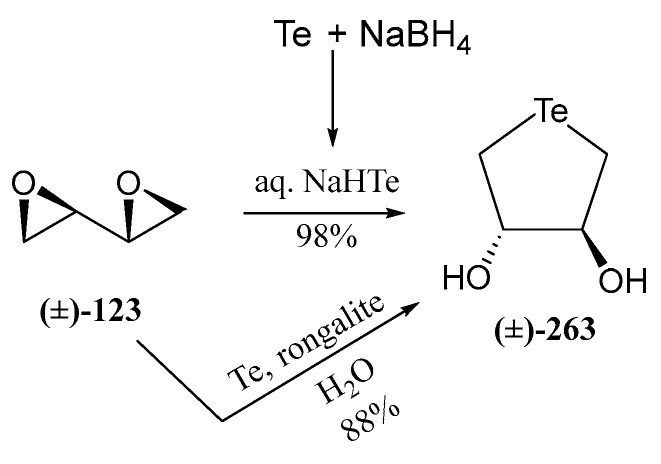
Preparation of *trans*-3,4-dihydroxy-1-tellurolane **263**.

Additionally, racemic *trans*-3,4-dihydroxy-1-tellurolane **263** was obtained [11] in almost quantitative yield by nucleophilic reaction of buta-1,3-diene bisepoxide **123** with aq. NaHTe (Scheme 47). Alternatively, Capperucci and coworkers reported [165] the synthesis of 263 under *on water* conditions and using rongalite (sodium hydroxymethanesulfinate) to reduce in situ elemental Te to Na_2_Te as the nucleophile (Scheme 47). Compound **263** is the Te-isoster of DHS^red^ 124, a selenosugar mimetics endowed with strong antioxidant properties, as considered in the precedent section.

**Scheme 48:**
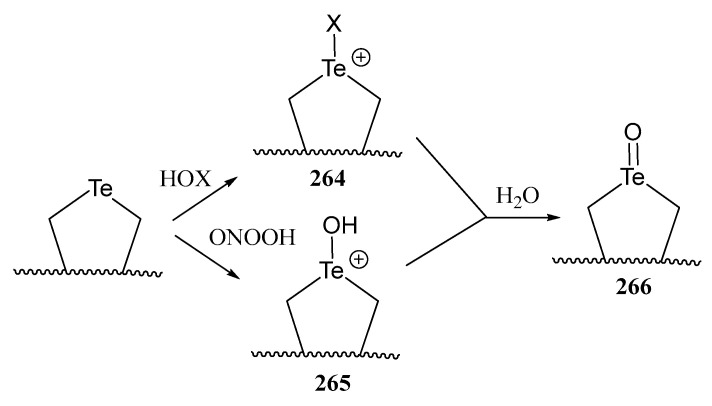
Proposed mechanism for the scavenging properties of tellurosugars against hypohalous and hyponitrous acids.

Tellurosugars **252**, **256**, **257**, **259** and **263** were assayed [11] for their scavenging properties against common oxidant agents like hypochlorous, hypobromous and peroxynitrous acids (HOCl, HOBr and ONOOH, respectively), generated by the inflammatory enzyme myeloperoxidase. Antioxidant properties against HOCl and HOBr were determined using a competition kinetics assay, while stopped-flow spectroscopy was used for monitoring reaction with ONOOH. 3,4-Dihydroxy-1-tellurolane **263** exhibited the best antioxidant profile against HOCl and ONOOH, whereas 4-tellurofuranose **259** was the best scavenger against HOBr [11]. A wider range of activities towards ONOOH was found among the set of compounds compared to hypohalous acids. The authors postulated the mechanism depicted in Scheme 48 for explaining the antioxidant properties of tellurosugars tested; it was suggested that such glycomimetics underwent oxidation to furnish telluroxide **266** through intermediates **264** (for hypohalous acids) and **265** (for hyponitrous acid).

In silico studies conducted on an ample number of seleno- and tellurosugars demonstrated that the incorporation of heavier chalcogen atoms (Se, Te) improves the antiradical properties of these glycomimetics compared to their sulfur isosters [166].

### 4.2. Telluroglycosides

**Scheme 49:**
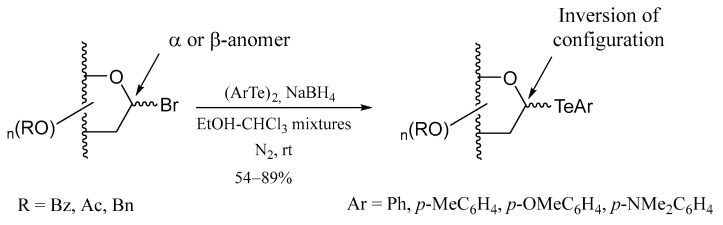
Preparation of telluroglycosides.

Incorporation of Te on the aglycon residue of a carbohydrate can be accomplished by reaction of *O*-protected (Bz, Ac, Bn) glycosyl bromides with diaryl ditellurides in the presence of NaBH_4_ [167]. Reactions proceeded smoothy, with good yields, and total stereospecificity; this outcome is compatible with an S_N_2-type reaction between the glycosyl bromide and the in situ generated aryl telluride, leading to the inversion of the configuration of the anomeric carbon (Scheme 49).

**Scheme 50:**
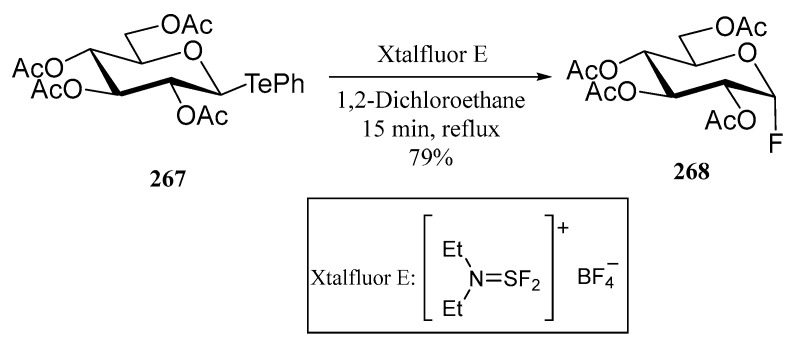
Preparation of glycosyl fluorides from telluroglycosides.

A relevant application of aryl telluro glycosides is their transformation into glycosyl fluorides, which are important tools in glycobiology, acting as probes in mechanistic studies of glycosidases and glycosyl transferases, and are also key intermediates for synthetic and enzymatic glycosylation reactions. Williams and coworkers reported [168] the straightforward transformation of thio-, seleno- and telluroglycosides into the corresponding glycosyl fluorides **268** using Xtalfluor reagents (E^®^ and M^®^) as an alternative to DAST and DAST/NBS as fluorine sources (Scheme 50). DAST is moisture sensitive and thermally unstable.

### 4.3. Sugar-Derived α-Alkoxyacyl Tellurides as Synthetic Intermediates

Inoue and coworkers developed [169] a robust convergent methodology for accessing complex polyoxygenated backbones featured with a high density of stereocenters. Such structure is frequently present in natural products, not only in saccharides, but also in secondary metabolites with great pharmaceutical interest. This methodology is based on a decarbonylative radical coupling reaction between sugar-derived α-alkoxyacyl tellurides and double bonds from aldehydes [170,171], oximes [169,172], enones [169,173], and *N*-heteroarenes [174]; homocoupling reactions between α-alkoxy radicals derived from carbohydrates have also been described [175,176].

**Scheme 51:**
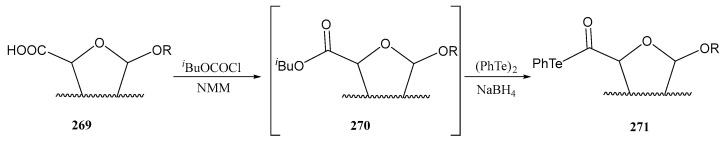
General synthetic pathway for the preparation of α-alkoxyacyl tellurides derived from carbohydrates.

α-Alkoxyacyl tellurides derived from carbohydrates (**271**) can be accessed [170] following the synthetic methodology depicted in Scheme 51. Thus, *O*-protected uronic acids **269** are converted into transient, non-isolated isobutyl esters **270** by treatment with isobutyl chloroformate in the presence of *N*-methylmorpholine (NMM). Subsequent reaction with in situ generated phenyl telluride (reduction of diphenyl ditelluride with NaBH_4_) afforded the corresponding α-alkoxyacyl telluride **271**.

**Scheme 52:**
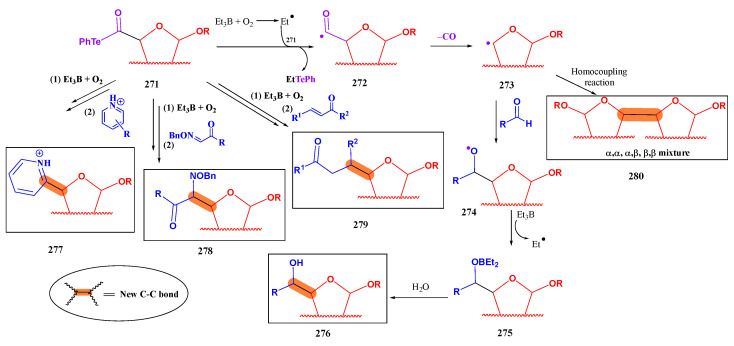
Proposed mechanism for the reaction of α-alkoxyacyl tellurides with radical acceptors.

Then, treatment of **271** with Et_3_B and O_2_ promotes the formation of an ethyl radical which in turn reacts with the organotellurium derivative to furnish an acyl radical (Scheme 52). This intermediate spontaneously undergoes a decarbonylation reaction, with the loss of CO to give an α-alkoxy radical, which is trapped by the acceptor (aldehyde, oxime, enone, or an *N*-heteroarene) to give derivatives **276**–**279** [171]; the complete mechanism is depicted, as a model process for coupling with aldehydes. Reaction with simple benzaldehyde failed, presumably due to the reduced electrophilicity of the carbonyl group because of conjugation with the aromatic residue [171]. Replacement of benzaldehyde with *o*-hydroxybenzaldedyde, and addition of a Lewis acid (Et_2_AlCl) successfully allowed the radical coupling reaction. It was claimed that complexation with the Lewis acid enhanced its reactivity. Radical-radical homocoupling reaction (without the addition of a free radical trapping reagent) [175] yielded dimers **280** as a mixture of diastereoisomers (Scheme 52). Coupling reactions between two different carbohydrate-derived radicals have also been reported [176].

**Figure 19:**
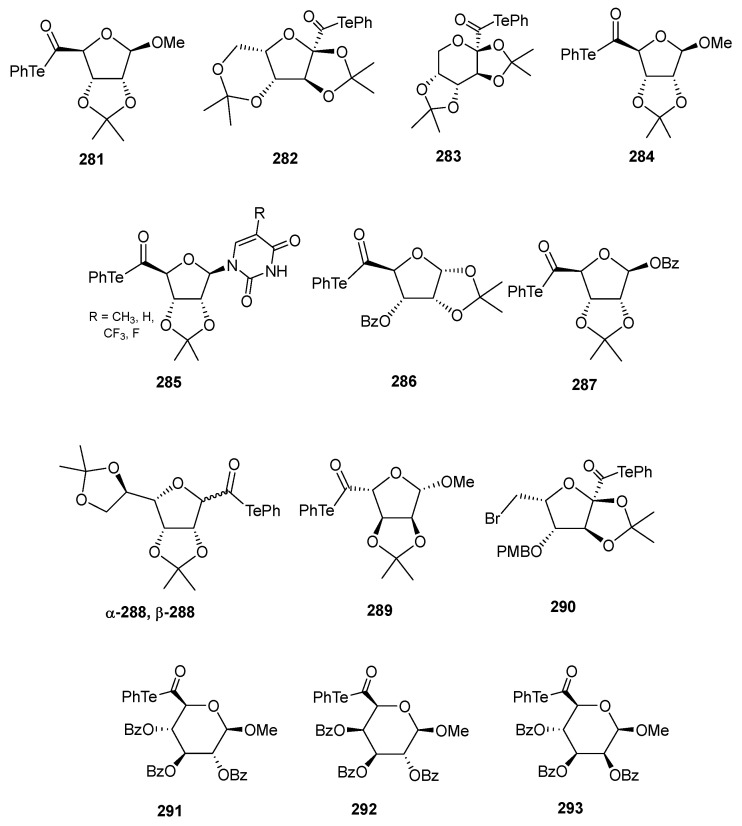
Sugar-derived α-alkoxyacyl tellurides as intermediates in free-radical reactions.

Figure 19 depicts the α-alkoxyacyl tellurides derived from carbohydrates that have been employed as key synthetic intermediates in the preparation of complex compounds, including natural products: **281**–**283** [169,171,174], **284**, **285** [172], **286** [171], **287**, **288** [175,176], **289** [170], **290** [173], **291**–**293** [175].

**Figure 20:**
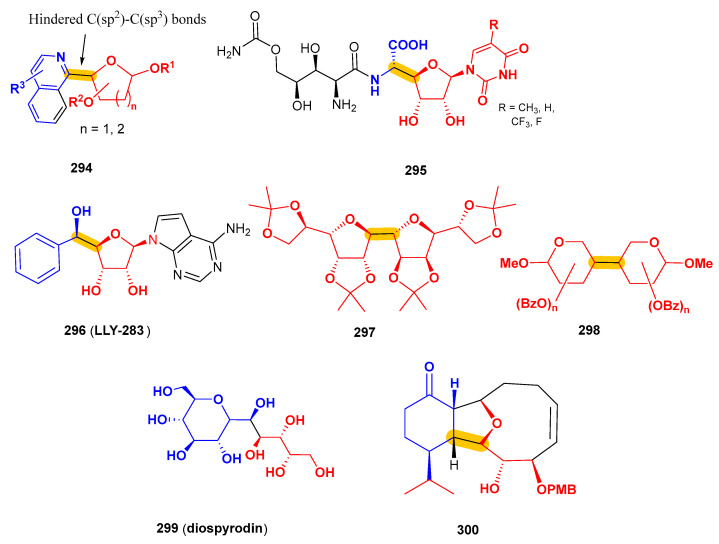
Examples of structures obtained from α-alkoxyacyl tellurides via free-radical reactions as one of the key steps.

Figure 20 shows a summary of the most relevant complex derivatives obtained from α-alkoxyacyl tellurides as one of the key intermediates: carbohydrate-*N*-heteroarene adducts through a hindered C(sp^2^)-C(sp^3^) junction (**294**) [174]; polyoxins J and L and fluoranalogues (**295**) [172]; the antitumour agent **LLY-283** (**296**) [171]; the C_2_-symmetric bis-tetrahydrofuran **297 [176]**, a template that is present in the skeleton of the natural anticancer agent asimicin, as well as dimeric pyranose scaffolds **298** [175], which may be suitable for the construction of polyoxygenated structures commonly found in natural bioactive compounds; the antibacterial agent diospyrodin (**299**) [170]; or the tricyclic derivative **300** [173], present in the skeleton of bioactive cladiellin diterpenoids.

## 5. Conclusions

In conclusion, the enormous importance of natural carbohydrates in a countless number of biochemical pathways has driven the development of glycomimetics to modulate or enhance their chemo-biological properties. Among the vast number of families of glycomimetics, those derived from the isosteric replacement of oxygen atoms at endocyclic or glycosidic positions by chalcogens (S, Se, Te) has received special attention. This transformation has paved the way for the synthesis of derivatives with unique conformational properties, increased hydrolytic stability, notable redox behavior, improved enzyme or receptor binding, and promising pharmacological effects. The growing interest in these compounds has also led to significant advances in the development of highly chemo- and regioselective reactions, environmentally friendly processes, chemoenzymatic synthesis, and even automated methodologies.

## Abbreviations

The following abbreviations are used in this manuscript:

A3AR	A3 Adenosine Receptor
AllNAc	N-Acetylallosamine
B2pin2	Bis(pinacolato)diboron
BSA	*N*,*O*-bis(trimethylsilyl)acetamide
BTD	Benzo-2,1,3-thiadiazole
C. diff.	Clostridioides difficile
Cer	Ceramide
CuAAC	Cu(I)-Catalyzed-Azide-Alkyne Cycloaddition
DBU	1,8-Diazabicyclo(5.4.0)undec-7-ene
Di-tBubpy	4,4-Di-tert-butyl-2,2–dipyridyl
DAST	Diethylaminosulfur trifluoride
DCM	Dichloromethane
DENV	Dengue Virus
DHS	Dihydroxy selenolane
DIAD	Diisopropyl azodicarboxylate
DMAP	4-(*N*,*N*-Dimethylamino)pyridine
DNsNH	2,4-(Dinitrobenzenesulfonyl)amino
DPAP	2,2-Dimethoxy-2-phenylacetophenone
DPPA	Diphenylphosphoryl azide
DTT	Dithiothreitol
EDA	Electron-Donor-Aceptor
Fdx	Fidaxomicin
GHS	Glutahione
GLUT	Glucose Transporters
GPx	Glutathione peroxidase
HCV	Hepatitis C Virus
HEMA	Poly(hydroxyethyl methacrylate)
HFIP	Hexafluoroisopropanol
HOMO	Highest Occupied Molecular Orbital
LED	Light Emitting Diode
LOOH	Lipid peroxide
MCP-1	Monocyte chemoattractant protein-1
MGL	Metabolic Glycan Labelling
MIC	Minimum Inhibitory Concentration
MPO	Myeloperoxidase
MsOH	Methanesulfonic acid
MUC1	Mucin 1
NBS	*N*-Bromosuccinimide
NDI	Naphthalene diimide
NHC	*N*-Heterocyclic carbene
NIS	*N*-Iodosuccinimide
NMM	*N*-Methylmorpholine
OGA	O-GlcNAcase
PDI	Protein disulfide isomerases
PEGDA	Poly(ethyleneglycol diacrylate)
PPAR	Peroxisome Proliferator-Activated Receptor
PTA	Phosphotungstic acid
*p*-QMS	*p*-Quinone methides
RBCs	Red Blood Cells
ROS	Reactive Oxygen Species
SET	Single Electron Transfer
Skp2	S-Phase Kinase Associated Protein 2
TBDPS	Terc-Butyldiphenylsilyl
TEMPO	2,2,6,6-Tetramethylpiperidine-1-oxyl, free radical
TIPDS	Tetraisopropyldisiloxanyl
TMSOTf	Trimethylsilyl triflate
TT	Thianthrenium
WGA	Wheat Germ Agglutin
XantPhos	9,9-Dimethyl-9H-xanthene-4,5-diyl)bis(diphenylphosphane
